# Screening of Fermentative Strains for Reducing the Allergenicity of a Whey Protein–Soy Protein System and Genomic Characterization of the Selected Strain

**DOI:** 10.3390/foods15172947

**Published:** 2026-08-22

**Authors:** Yunlei Chai, Qinggang Xie, Qingfeng Zhang, Guisong Bai, Yujun Jiang, Ling Guo, Yu Zhang, Jianguo Sun, Junqing Zhang

**Affiliations:** 1 Key Laboratory of Dairy Science, Ministry of Education, College of Food Science, Northeast Agricultural University, Harbin 150030, China; 2 Heilongjiang Feihe Dairy Co., Ltd., Qiqihar 164800, China; 3 Key Laboratory of Infant Formula Food, State Administration for Market Regulation, Harbin 150030, China

**Keywords:** whey protein isolate, soy protein isolate, *Lacticaseibacillus paracasei*, allergenicity, whole genome analysis

## Abstract

Dual-protein systems combining whey protein isolate (WPI) and soy protein isolate (SPI) offer complementary nutritional benefits but are limited by the presence of major allergens. Lactic acid bacteria (LAB) fermentation provides a promising strategy to mitigate this limitation. In this study, *Lacticaseibacillus paracasei* JM053, selected from 13 LAB strains based on phenotypic screening, significantly reduced the in vitro allergenicity of the dual-protein system, increasing the IgE-binding inhibition rate to 48.75%. Whole-genome sequencing and characterization of JM053 revealed a comprehensive proteolytic system, including the proline-specific peptidase genes *pepX* and *pepQ*, which may contribute to the degradation of allergenic peptide sequences. Combined with in silico bioinformatic analysis, potential cleavage sites within the linear epitopes of the dual-protein system were predicted based on the substrate specificity of the identified proteases, offering a testable hypothesis for the strain’s mechanism of action. In addition, in vitro safety assessment and genomic analysis supported the safety potential, stress tolerance, and probiotic characteristics of JM053. Collectively, this study provides a valuable candidate strain for the development of hypoallergenic dual-protein products and offers preliminary genomic insights into LAB-mediated allergenicity reduction.

## 1. Introduction

Proteins are essential nutrients for sustaining human physiological functions and serve as core functional ingredients in the food industry [1,2,3,4]. With the growing consumer demand for nutritious and healthy foods, the limitations of single-source proteins have become increasingly apparent. Whey protein isolate (WPI) and soy protein isolate (SPI) are representative high-quality animal and plant proteins, respectively. However, WPI is often limited by resource availability and high production costs [5], whereas SPI, despite being abundant and cost-effective, is deficient in certain essential amino acids and contains anti-nutritional factors, thus failing to fully meet human nutritional requirements [6]. Therefore, from both nutritional and economic perspectives, incorporating WPI and SPI into a dual-protein system not only alleviates supply pressures and optimizes amino acid profiles, but also offers the potential to enhance protein digestibility and bioavailability [7].

However, β-lactoglobulin (β-Lg) and α-lactalbumin (α-La) in WPI, together with glycinin (11S) and β-conglycinin (7S) in SPI, are widely recognized as allergenic proteins [8,9]. Ingestion of these proteins can trigger adverse reactions in sensitive individuals, ranging from skin rashes and respiratory distress to gastrointestinal dysfunction, and in severe cases, life-threatening anaphylactic shock [10]. Consequently, combining WPI and SPI may broaden the spectrum of the allergic population, thereby severely restricting the wider industrial application and market promotion of such dual-protein formulations. Therefore, exploring effective strategies to reduce the allergenicity of the WPI-SPI dual-protein system is of paramount practical significance for the development of hypoallergenic or non-allergenic dual-protein functional foods.

The allergenicity of SPI and WPI is closely associated with their linear and conformational epitopes, which can be modified through various processing techniques, including heating, enzymatic hydrolysis, high-pressure processing, ultrasound treatment, covalent polyphenolic conjugation, and microbial fermentation [11,12]. Lactic acid bacteria (LAB) fermentation, as a green and mild biological modification technique, has been widely applied in research on the reduction in protein allergenicity [13,14,15,16]. *Lactiplantibacillus plantarum* AHQ-14, a strain originally isolated from Xinjiang fermented dairy products, exhibited significant inhibitory effects on the IgE-binding capacity of α-La, β-Lg, and α-casein (α-CN), with the lowest allergenicity of these proteins observed after 10 h of cultivation [17]. *Clostridium tyrobutyricum* Z816 effectively degraded β-Lg antigenic epitopes, and studies indicated that optimized environmental conditions could further enhance its strain activity and functional expression [18]. Du et al. [19] discovered that *Pediococcus pentosaceus* C1001, *Pediococcus acidilactici* E1601-1, and *Lacticaseibacillus paracasei* E1601-2 effectively hydrolyzed β-Lg, thereby reducing its allergenicity by over 40%. Among these strains, *L. paracasei* E1601-2 possesses a relatively complete proteolytic system and is capable of degrading a broader range of allergenic peptides. Similarly, fermentation of the soybean 7S subunit with *L. plantarum* B1-6 significantly reduced its antigenicity, with the lowest antigenicity of 7S observed at a fermentation endpoint pH of 4.5 [20]. Mixed solid-state fermentation using *Lacticaseibacillus casei*, *yeast*, and *Bacillus subtilis* also effectively decreased soybean meal allergenicity [21]. However, studies on the hydrolysis and allergenicity of WPI-SPI dual-protein systems by LAB fermentation remain scarce. LAB possess a complex proteolytic enzyme system composed of cell envelope proteinases (CEPs), protein transport systems, and intracellular peptidases. During fermentation, these proteases degrade proteins into peptides and amino acids, thereby destroying or masking allergenic epitopes and consequently altering antigenicity [22]. To date, the mechanisms underlying protease-mediated desensitization by LAB at the genomic level have not yet been elucidated.

Based on this background, this study evaluated 13 LAB strains using multiple indicators—including degree of hydrolysis, peptide concentration, free amino acid release, antigenicity, and IgE-binding inhibition rate—to identify a promising strain for reducing the allergenicity of the WPI-SPI dual-protein system. Whole-genome sequencing of the selected strain was then conducted to characterize its proteolysis-related genetic features and to provide preliminary insights into its underlying mechanism of allergenicity reduction. Additionally, the safety profile of the selected strain was assessed through in vitro assays and genomic analysis. Overall, this study offers a candidate strain and valuable data to support the development of hypoallergenic dual-protein ingredients.

## 2. Materials and Methods

### 2.1. Materials and Reagents

WPI (9410, 90%) was obtained from Hilmar Ingredients (Hilmar, CA, USA). SPI (90%) was obtained from Wonderful Biotechnology Co., Ltd. (Dongying, China). Goat anti-human immunoglobulin E (IgE)-horseradish peroxidase (HRP) was obtained from Bioss Biotechnology Co., Ltd. (Beijing, China). Serum samples from 6 patients with cow’s milk and soybean allergies (Table S1) were obtained from Wolcavi Biotechnology Co., Ltd. (Chongqing, China). Equal volumes of individual serum samples were mixed to prepare a pooled serum for later use.

### 2.2. Strain Preparation

A total of 13 LAB strains were used in this study, all of which were maintained at the Key Laboratory of Dairy Science, Northeast Agricultural University. These strains were selected from our laboratory collection based on literature reports of proteolytic or allergenicity-reducing potential, as well as prior in-house data confirming their multiple beneficial functions and safety [23,24,25,26,27,28,29,30,31]. Detailed information, including selection criteria for each strain, is provided in Table S2. The selected strains were introduced into de Man, Rogosa, and Sharpe (MRS) broth at 4% (*v*/*v*) and cultivated at 37 °C for 20 h. After activation, a loopful of the culture was inoculated onto MRS agar and held at 37 °C for 24 h. One colony was then introduced into MRS broth and cultured at 37 °C for 20 h [32]. The bacterial suspension was subjected to DNA extraction and 16S rDNA sequencing for strain identification. Following identity confirmation, the strains were used in experimental studies. The activated culture was supplemented with glycerol (80% *v*/*v*) to a final concentration of 25% (*v*/*v*) and maintained at −80 °C for long-term storage.

### 2.3. Preparation of Fermented Dual-Protein

WPI and SPI were mixed at a 1:1 ratio to give a 5% (*w*/*v*) solution. The pH was adjusted to 7.0, stirred for 2 h, and then refrigerated at 4 °C overnight for hydration. The next day, glucose was added to 2% (*w*/*v*) and stirred until homogeneous [33]. After heating at 95 °C for 10 min in a water bath, the solution was cooled to room temperature and stored for later use.

The activated bacterial suspension was centrifuged, and the pellet was washed twice with sterile saline and resuspended in the same saline to obtain a working suspension. The dual-protein solution was inoculated with the suspension at 4% (*v*/*v*) to achieve an initial viable cell concentration of 10^7^ CFU/mL, and then fermented for 48 h at 37 °C, followed by centrifugation; the supernatant was kept for analysis [34]. Prior to analysis, all supernatants were adjusted with ultrapure water to an identical total soluble protein concentration to exclude the interference of protein solubility variations. The dual-protein samples before and after fermentation were designated WPI-SPI and FWPI-SPI, respectively.

### 2.4. Screening for Hypoallergenic Strains

#### 2.4.1. Determination of pH and Viable Cell Counts

Viable cell counts were determined using the standard plate-counting method and expressed as CFU/mL. The pH of the samples was measured using a Delta 320 pH meter (Mettler-Toledo International Inc., Shanghai, China). All analyses were performed in triplicate [34].

#### 2.4.2. Determination of Protease Activity

Protease activity was measured following the protocol outlined by Yuan et al. [35], with slight modifications. Briefly, 200 μL of fermentation supernatant was mixed with 500 μL of 0.2% azocasein and incubated at 37 °C for 30 min. The reaction was stopped with 10% trichloroacetic acid (TCA). After centrifugation at 12,000 rpm for 10 min, the absorbance of the supernatant was measured at 366 nm, with phosphate-buffered saline (PBS) as the blank. One unit (U) of protease activity corresponded to the amount of enzyme required to increase the absorbance at 366 nm by 0.01 per hour at 37 °C.

#### 2.4.3. Degree of Hydrolysis (DH)

The degree of protein hydrolysis was quantified using the OPA method with minor adjustments of Dong et al. [32]. Briefly, 400 μL of the protein digest was mixed with 3 mL of OPA reagent, and the mixture was incubated at ambient temperature in the dark for 2 min. Subsequently, the absorbance was measured at 340 nm. A serine standard solution and PBS were used as the standard and blank control, respectively.

#### 2.4.4. Peptide Concentration

The peptide concentration was determined using the BeyoBCA Peptide Concentration Assay Kit (Shanghai Beyotime Biotechnology Co., Ltd., Shanghai, China) with slight modifications [36]. Briefly, the post-fermentation protein supernatant was collected and mixed with a 10% TCA solution (1:1, *v*/*v*). After incubation for 30 min and subsequent centrifugation, the supernatant was transferred to a Millipore ultrafiltration centrifugal tube (<10 kDa) and centrifuged at 3000× *g* for an additional 15 min at 4 °C. The resulting filtrate was used for subsequent analysis.

#### 2.4.5. Free Amino Acid

Free amino acids were quantified following a slightly modified method from Dong et al. [32]. Briefly, the post-fermentation protein supernatant was mixed with an equal volume of 5% sulfosalicylic acid solution and kept at ambient temperature for 1 h. After centrifugation, the supernatant was collected, stored at 4 °C overnight, and then passed through a 0.22 μm membrane. The resulting filtrate was subsequently assessed for free amino acid composition using a Hitachi LA8080 amino acid analyzer (Hitachi High-Tech Corp., Tokyo, Japan).

#### 2.4.6. SDS-PAGE and Tricine-SDS-PAGE

Sodium dodecyl sulfate-polyacrylamide gel electrophoresis (SDS-PAGE) was performed according to Pang et al. [37], using a commercial kit (Shanghai Yamei Biomedical Technology Co., Ltd., Shanghai, China) featuring a 4.5% stacking gel and a 15% resolving gel. Samples (1 mg/mL) were mixed with the loading buffer at a 1:1 ratio (*v*/*v*) and heated at 100 °C for 5 min. The loading volume was 10 μL. Electrophoresis was initially conducted at 80 V until the samples entered the resolving gel, after which the voltage was increased to 120 V. After electrophoresis, the gel was stained with Coomassie Brilliant Blue G-250 for 30 min and then destained with a destaining solution until clear protein bands were visible against a transparent background. Subsequently, the gel was imaged using a Bio-Rad gel imaging system (Bio-Rad Laboratories, Hercules, CA, USA).

Tricine-SDS-PAGE was carried out using a commercial kit (Shanghai Yamei Biomedical Technology Co., Ltd., Shanghai, China) to analyze the low-molecular-weight peptides (1–10 kDa) generated after dual-protein fermentation. A 4.5% stacking gel and a 16% resolving gel were prepared, followed by electrophoresis at 120 V for 2 h [38].

#### 2.4.7. Determination of Antigenicity

Changes in the antigenicity of allergenic proteins in the fermentation broth were determined using an indirect ELISA. Determinations were performed using soybean 7S, soybean 11S, bovine α-La, and bovine β-Lg ELISA Kits (Jiangsu Enzyme Immunoassay Industry Co., Ltd., Yancheng, China), respectively [39].

$$\mathrm{Antigenicity}(\%)=\frac{\mathrm{Antigen}\;\mathrm{concentration}\;\mathrm{of}\;\mathrm{the}\;\mathrm{adjusted}\;\mathrm{FWPI}-\mathrm{SPI}}{\mathrm{Antigen}\;\mathrm{concentration}\;\mathrm{of}\;\mathrm{the}\;\mathrm{adjusted}\;\mathrm{WPI}-\mathrm{SPI}}\;\times \;100$$

#### 2.4.8. Determination of IgE-Binding Inhibition Rate

The IgE-binding capacity of fermented proteins was determined via indirect ELISA using allergic human sera according to the methods of Pang et al. [37]. Samples (100 μL, 2 μg/mL in 0.05 M sodium carbonate buffer, pH 9.6) were coated onto 96-well plates at 4 °C overnight. After blocking with 2.5% bovine serum albumin (BSA), the wells were sequentially incubated with diluted serum samples and HRP-conjugated goat anti-human IgE (each for 1 h at 37 °C), with PBST (10 mM PBS, pH 7.5, 0.05% Tween-20) washes between incubation steps. Subsequently, 3,3′,5,5′-tetramethylbenzidine (TMB) substrate was added and incubated for 15 min at 37 °C in the dark. The reaction was terminated by adding 2 M H_2_SO_4_, and the absorbance was measured at 450 nm. IgE-binding capacity was calculated as follows:

$$IgE-binding\;inhibition\;rate\;(\%)=(1-\frac{{\mathrm{OD}}_{450}\left(\mathrm{FWPI}-\mathrm{SPI}\right)}{{\mathrm{OD}}_{450}\left(\mathrm{WPI}-\mathrm{SPI}\right)})\times 100$$

### 2.5. In Vitro Safety Evaluation of the Selected LAB

#### 2.5.1. Antibiotic Resistance Assay

Antimicrobial susceptibility of *Lacticaseibacillus paracasei* strain JM053 was assessed by broth microdilution MIC determination according to ISO 10932:2010 and EFSA guidelines [40,41]. *L. paracasei* ATCC 334 was included as the quality-control strain, and its MIC values were evaluated against the acceptable ranges reported based on ISO 10932 [42]. Eight antibiotics (Ampicillin, Gentamicin, Kanamycin, Streptomycin, Erythromycin, Clindamycin, Tetracycline, and Chloramphenicol) were tested. Bacterial suspensions were adjusted to 5 × 10^5^ CFU/mL and exposed to two-fold serial antibiotic dilutions. The MIC was defined as the lowest concentration that completely inhibited visible growth, and susceptibility was classified by comparison with EFSA species-specific cut-off values for *L. paracasei*.

#### 2.5.2. Hemolytic Assay

*L. paracasei* JM053 and *Staphylococcus aureus* (*S. aureus*) ATCC 25923 were separately inoculated by streaking onto Columbia agar plates supplemented with 5% sterile defibrinated sheep blood, and then held for 48 h at 37 °C, after which the hemolytic patterns were examined: α-hemolysis appeared as a greenish halo around the colonies, β-hemolysis as a clear transparent zone, and γ-hemolysis (non-hemolytic) as the absence of any hemolytic zone [43].

#### 2.5.3. Indole Assay

*L. paracasei* JM053 and *E. coli* ATCC 25922 were individually introduced into peptone water, with uninoculated broth serving as the blank control. After 72 h of incubation at 37 °C, nine drops of Kovac’s indole reagent were added, and the color change was observed. The appearance of a red ring at the liquid–air interface indicated a positive indole test, whereas its absence denoted a negative one.

#### 2.5.4. Nitrate Reduction Assay

Nitrate broth was separately inoculated with *L. paracasei* JM053 and *E. coli* ATCC 25922, while uninoculated broth was used as a blank control. After a 5-day incubation at 37 °C, 10 drops each of 5% potassium iodide solution and 5% starch solution were introduced sequentially. The mixture was examined for a color change: the formation of blue was regarded as a positive result, whereas the absence of blue was considered negative [32].

#### 2.5.5. Amino Acid Decarboxylase Assay

Broths for arginine dihydrolase, lysine decarboxylase, and ornithine decarboxylase were separately inoculated with *L. paracasei* JM053 and *E. coli* ATCC 25922. Each inoculated broth was overlaid with 1 cm of sterile liquid paraffin, followed by incubation at 37 °C for 72 h, after which a purple color was considered positive and a yellow color negative [44].

### 2.6. Whole-Genome Analysis

The complete genome of *L. paracasei* JM053 was sequenced using a combination of the Illumina HiSeq and PacBio platforms. The raw sequencing data were quality-controlled and filtered using fastp (v0.23.1). Subsequent sequence correction and de novo assembly were performed using Pilon (v1.24), Unicycler (v0.5.0), Flye (v2.9.1), Hifiasm (v0.18.5), and Necat (v0.0.1) to obtain the complete genome sequence (Shanghai Personal Biotechnology Co., Ltd., Shanghai, China). Protein-coding genes were predicted using GeneMarkS (v4.32). Genes associated with proteolytic activity were identified and analyzed through functional annotation against the eggNOG, GO, and KEGG databases. Carbohydrate-active enzymes were annotated using the Carbohydrate-Active Enzymes database (CAZy, http://www.cazy.org/, accessed on 22 August 2026). Biosynthetic gene clusters for secondary metabolites were identified using antiSMASH (v7.1.0). Virulence factors and antibiotic resistance genes were identified using the Virulence Factor Database (VFDB, http://www.mgc.ac.cn/VFs/, accessed on 22 August 2026) and the Comprehensive Antibiotic Resistance Database (CARD, https://card.mcmaster.ca/, accessed on 22 August 2026), respectively. The complete genome sequence of *L. paracasei* JM053 has been deposited in the National Center for Biotechnology Information (NCBI) GenBank database under the accession number JBWQQG000000000.

### 2.7. Statistical Analysis

All experiments were performed with three independent biological replicates, and results are expressed as mean ± SD. One-way ANOVA was performed using IBM SPSS Statistics 27.0.1, with a significance threshold of *p* < 0.05. Graphical presentations were prepared using Origin 2024.

## 3. Results and Discussion

### 3.1. Results of Strain Screening

#### 3.1.1. pH and Viable Cell Counts

As shown in Table 1, after 48 h of fermentation in WPI-SPI, the pH values of all fermented samples decreased significantly, ranging from 3.58 to 4.56. The viable counts after 48 h ranged from 4.0 × 10^6^ to 7.8 × 10^6^ CFU/mL, which were lower than the initial inoculum level, indicating a decline in viable cell numbers during fermentation. The decrease in pH was mainly attributed to organic acid production during carbohydrate metabolism [34]. Although no direct correlation was observed between final pH and viable counts, the reduction in viable cell numbers may be partially related to prolonged exposure to acidic conditions during fermentation [45]. Notably, *L. paracasei* JM053 showed a viable count of 7.4 × 10^6^ CFU/mL at a final pH of 3.77, which was relatively high among the tested strains.

#### 3.1.2. Protease Activity

**Figure 1:**
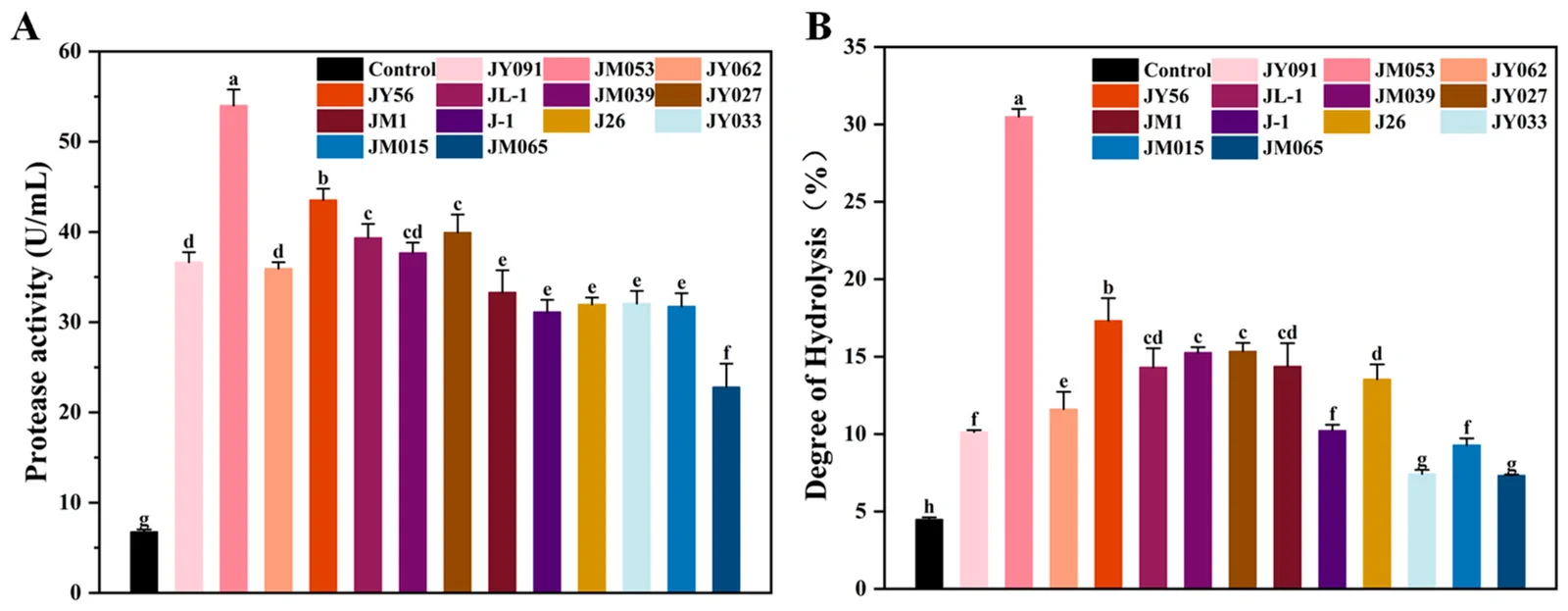
(**A**) Protease activity. (**B**) Degree of hydrolysis. The presence of different letters in the same picture serves to indicate significant differences (*p* < 0.05).

Protease activity in the culture supernatant, a direct indicator of proteolytic capacity, varied considerably among the tested strains (Figure 1A). Notably, *L. paracasei* JM053 exhibited the highest activity (54.01 U/mL), whereas *L. plantarum* JM065 showed the lowest (22.80 U/mL). The underlying reasons for this strain-dependent variation remain unclear, although differences in environmental adaptability and genetic background may play a role [11]. Further molecular studies are warranted to elucidate the mechanisms underlying these strain-dependent differences.

#### 3.1.3. DH

The DH is a key indicator for evaluating protein degradation. As illustrated in Figure 1B, compared with the WPI-SPI control, the DH of the FWPI-SPI was significantly increased across all tested strains, and *L. paracasei* JM053 exhibited the highest hydrolysis rate (30.50%) in the WPI-SPI dual protein system, which was significantly different from that of the other strains (*p* < 0.05). Proteases produced by lactic acid bacteria during fermentation can hydrolyze proteins into smaller peptides and amino acids, thereby increasing the degree of protein hydrolysis [46]. The increased DH reflects enhanced degradation of protein molecules, suggesting that proteolysis may alter the primary structure of proteins, which in turn could affect the integrity of antigenic epitopes [39,47]. Therefore, the enhanced hydrolysis ability of JM053 may have favorable implications for modifying protein antigenicity, warranting further investigation.

#### 3.1.4. Peptide Concentration and Free Amino Acids

**Figure 2:**
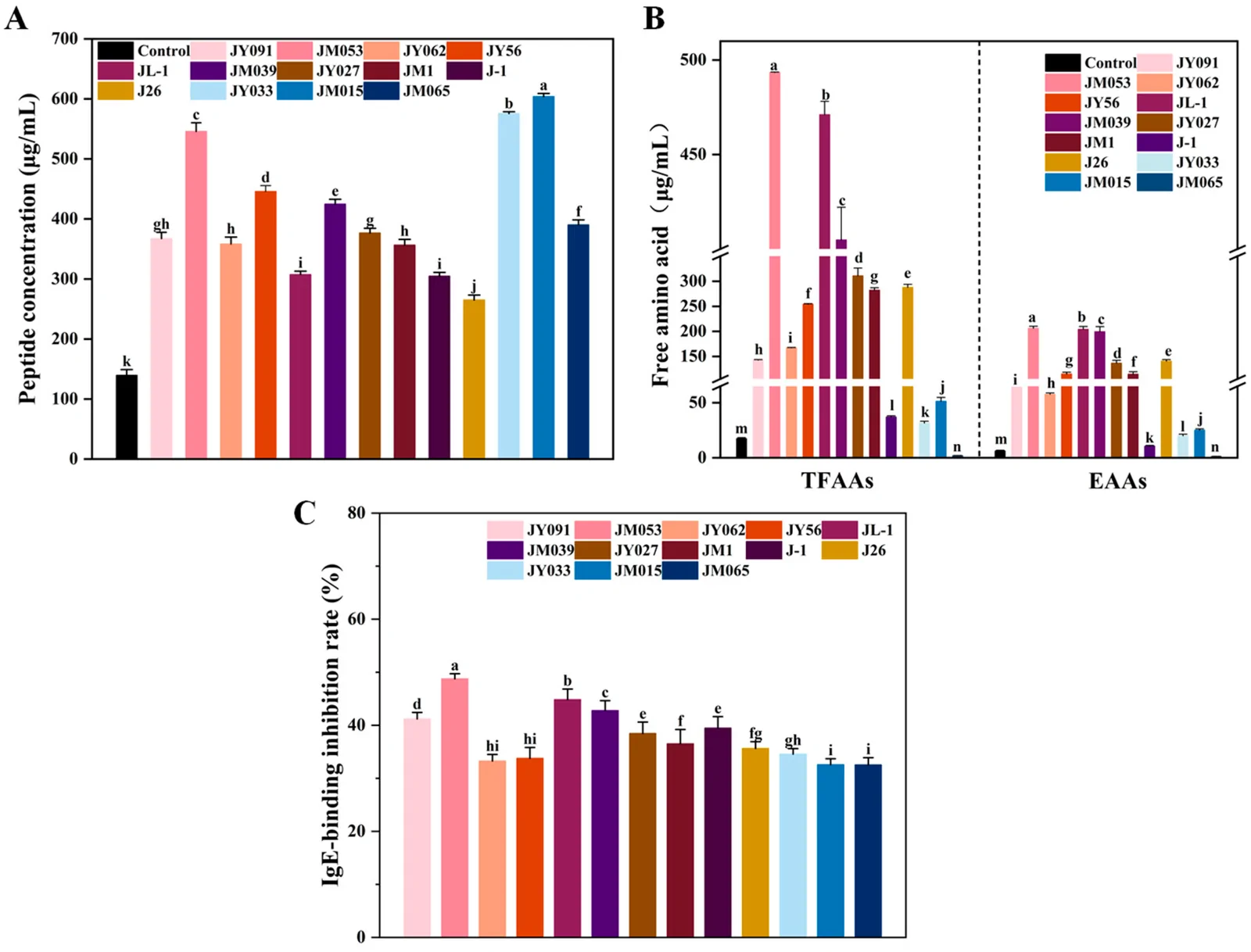
(**A**) Peptide concentration. (**B**) Free amino acid. (**C**) IgE-binding inhibition rate. The presence of different letters in the same picture serves to indicate significant differences (*p* < 0.05).

As illustrated in Figure 2A, the peptide concentration in the WPI-SPI dual-protein solutions fermented by the strains increased significantly compared to the unfermented control, with increments ranging from 125.5 to 464.4 μg/mL relative to the unfermented control (139.5 μg/mL). However, the trends in peptide concentration variations among different strains differed from the aforementioned results. For instance, although *L. paracasei* JM053 exhibited the highest protease activity and degree of hydrolysis, its fermented broth possessed a lower peptide concentration (545.8 μg/mL) than those of *L. plantarum* 015 (604.0 μg/mL) and JY033 (575.7 μg/mL). This discrepancy could be attributed to the dynamic accumulation and consumption of peptides during fermentation, which is governed by the synergistic action of proteases and peptidases [48]. Specifically, the peptides released via hydrolysis by JM053 might be further degraded into smaller molecules by its highly efficient peptidase system. This hypothesis was supported by the free amino acid (FAA) profile; following fermentation by JM053, the total FAA content reached 493.42 μg/mL, representing an approximately 28.7-fold increase compared to the unfermented control (17.22 μg/mL). Notably, JM053 also yielded the highest total essential amino acid (EAA) content (206.13 μg/mL) among all tested strains (Table 2 and Figure 2B). Collectively, these results suggest that JM053 exhibited the strongest overall proteolytic and peptidolytic performance among the tested strains, as reflected by enhanced protein hydrolysis and the subsequent conversion of peptides into free amino acids.

Notably, *L. paracasei* JM053 released the highest total levels of free amino acids (FAAs) associated with the allergenic epitopes of WPI and SPI. The key IgE-binding epitopes of α-La and β-Lg are rich in Val, Phe, Arg, Tyr, Leu, His and Asp, Met, Thr, respectively [49,50]. JM053 yielded the maximum total concentration of these WPI-related allergenic FAAs (188.14 µg/mL). Regarding SPI, whose 7S and 11S subunits are abundant in Asp/Asn, Glu/Gln, Leu, Arg, Lys, and His [51], fermentation with JM053 generated a total SPI-related allergenic FAA concentration of 207.34 µg/mL, which was second only to *L. rhamnosus* JL-1 (210.79 µg/mL). These strain-specific FAA profiles likely reflected distinct proteolytic and peptidolytic cleavage specificities among the tested strains, indicating that the enzymatic systems of different LAB strains exhibited preferential hydrolysis toward different protein substrates and peptide bonds.

#### 3.1.5. Electrophoresis

**Figure 3:**
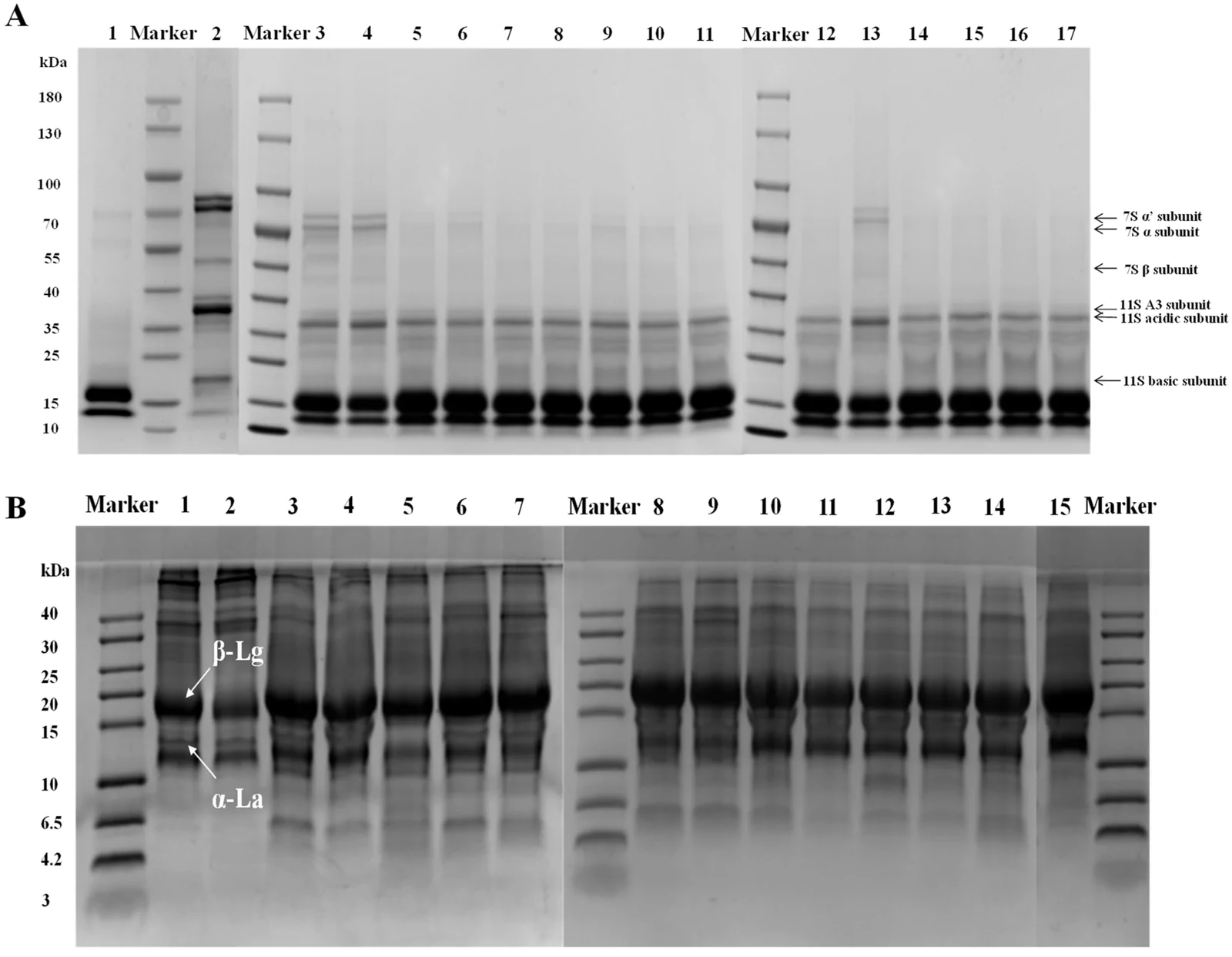
(**A**) SDS-PAGE, 1: WPI, 2: SPI, 3: WPI-SPI, 4: Heat-sterilized WPI-SPI, 5: JY091, 6: JM053, 7: JY062, 8: JY56, 9: JL-1, 10: JM039, 11: JY027, 12: JM1, 13: J-1, 14: JY033, 15: J26, 16: JM065, 17: JM015 (**B**) Tricine-SDS-PAGE, 1: WPI-SPI, 2: Heat-sterilized WPI-SPI, 3: JY091, 4: JY062, 5: JM053, 6: JY56, 7: JL-1, 8: JM039, 9: JY027, 10: JM1, 11: J-1, 12: JM065, 13: J26, 14: JY033, 15: JM015.

SDS-PAGE analysis of WPI, SPI, and WPI-SPI samples showed characteristic bands corresponding to α-La (~14 kDa), β-Lg (~18 kDa), and the 7S (α′, α, β) and 11S (A3, acidic and basic) subunits, consistent with previously reported patterns (Figure 3A) [34,37]. The WPI-SPI mixture exhibited a banding pattern similar to the individual proteins, with no additional bands detected. The heat-treated WPI-SPI sample showed a comparable profile, indicating that heat sterilization did not cause noticeable protein degradation. After fermentation, the 7S and 11S subunit bands were weakened or disappeared in all strains, while some strains generated a new band at approximately 24 kDa, suggesting partial degradation of high-molecular-weight proteins. Notably, fermentation with *L. paracasei* JM053 resulted in extensive degradation without the accumulation of intermediate bands, which may indicate more efficient hydrolysis into smaller peptides.

Tricine SDS-PAGE was performed to further characterize low-molecular-weight peptide profiles (Figure 3B). Similarly, the banding profiles of the WPI-SPI mixture before and after heating were comparable to those of the individual proteins. After fermentation, the β-Lg region became broader in all fermented samples, likely due to the accumulation of proteolytic fragments migrating in this region, whereas the broadening was less pronounced in the JM053 group. The α-La band remained largely intact in most fermented samples but was markedly reduced after fermentation with JM053. Notably, JM053 fermentation resulted in fewer intermediate bands between α-La and β-Lg and more abundant low-molecular-weight bands (<10 kDa), suggesting a greater extent of protein breakdown and an increased abundance of smaller peptide fragments in the WPI-SPI dual-protein system.

#### 3.1.6. Antigenicity

**Figure 4:**
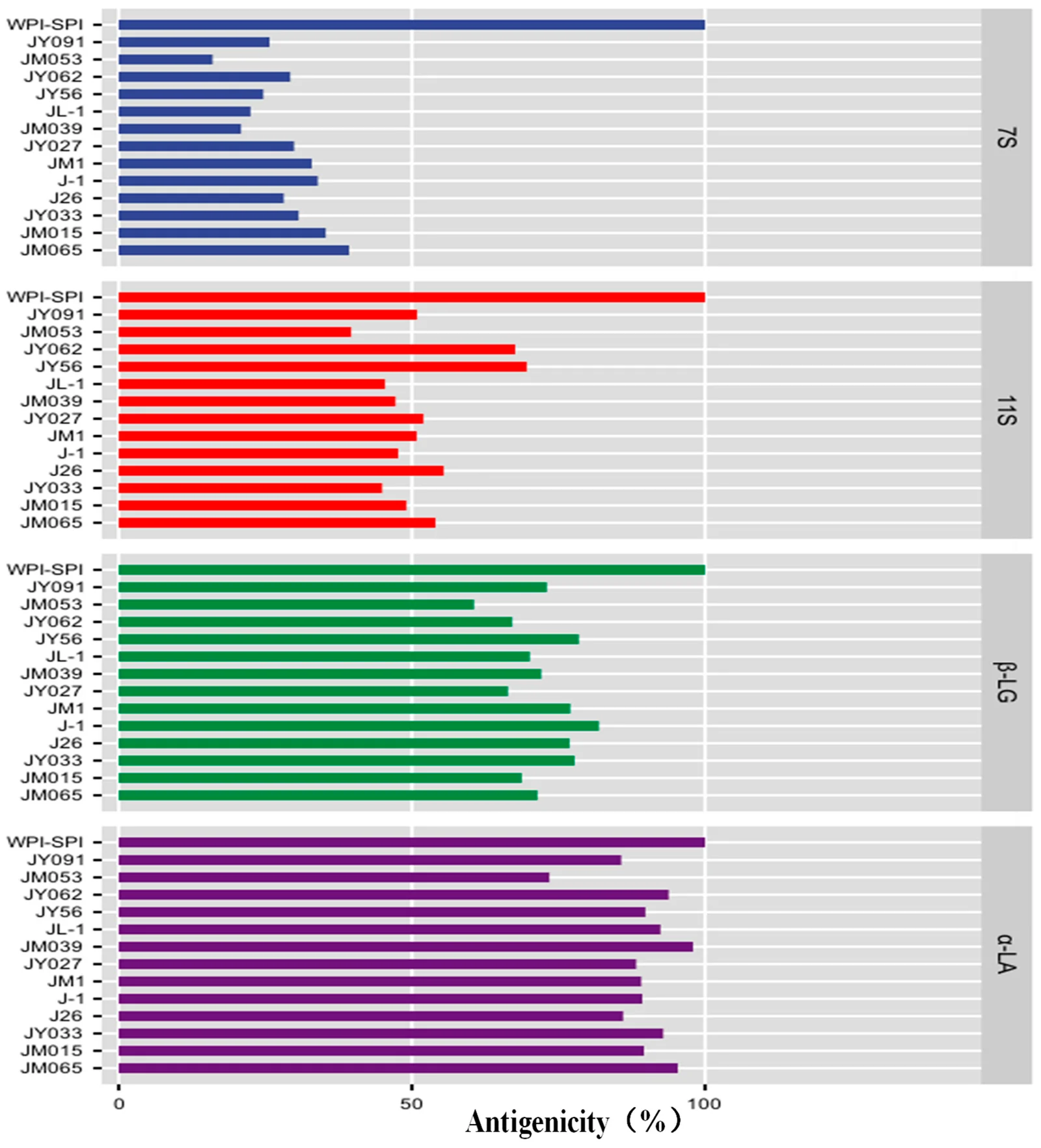
The Antigenicity of fermentation with different strains on the antigenicity of four proteins: 7S and 11S subunits in SPI, and whey α-La and β-Lg in WPI.

As shown in Figure 4, compared with the WPI-SPI control, the antigenicity of each allergenic protein in the FWPI-SPI was markedly reduced across all strains. The residual antigenicity ranked in the increasing order of: 7S (16.01–39.28%) < 11S (39.63–69.57%) < β-Lg (60.58–81.88%) < α-La (73.44–97.94%). This reduction may be associated with the hydrolysis of peptide bonds or conformational alterations induced by lactic acid bacteria proteolysis, which could disrupt or mask antigenic epitopes within WPI and SPI [39]. Notably, *L. paracasei* JM053 fermentation resulted in the lowest residual antigenicity for all four allergenic proteins, suggesting that JM053 exhibited the strongest reduction in antigenicity among the tested strains within the WPI-SPI dual-protein system.

#### 3.1.7. IgE-Binding Inhibition Rate

Allergic reactions induced by SPI and WPI are typical IgE-mediated type I hypersensitivity reactions. As shown in Figure 2C, the inhibition rate of IgE-binding for the FWPI-SPI mixtures varied among the tested strains, ranging from 32.54% to 48.75%. Similarly, enzymatic hydrolysis has been reported to disrupt conformational epitopes or cleave linear epitopes, which may enhance the inhibition rate of IgE-binding and thus potentially attenuate overall allergenicity [26,38]. Notably, fermentation with *L. paracasei* JM053 resulted in the highest inhibition rate (48.75%, *p* < 0.05) among all tested strains. Together with the proteolysis results mentioned above, these findings appear to suggest that fermentation with JM053 may contribute to reducing the allergenicity of the WPI-SPI dual-protein system. Nevertheless, while the elevated IgE-binding inhibition rate indicates reduced epitope accessibility, this in vitro binding assay only reflects changes at the antigen–antibody recognition level and cannot substitute for the assessment of effector cell functionality. Therefore, further degranulation assays would be necessary to confirm its clinical relevance.

### 3.2. In Vitro Safety Assessment of L. paracasei JM053

#### 3.2.1. Antibiotic Susceptibility

The minimum inhibitory concentrations (MICs) of a panel of antibiotics against *L. paracasei* JM053 were determined (Table S3). The MIC values of the quality-control strain *L. paracasei* ATCC 334 were within the acceptable ranges, supporting the validity of the assay. The MICs for JM053 were as follows: ampicillin, 1 μg/mL; gentamicin, 8 μg/mL; kanamycin, 32 μg/mL; streptomycin, 32 μg/mL; erythromycin, 0.5 μg/mL; clindamycin, 0.5 μg/mL; tetracycline, 0.25 μg/mL; and chloramphenicol, 2 μg/mL. All MIC values were below the species-specific microbiological cut-off values recommended by EFSA, suggesting that JM053 showed no phenotypic resistance to the tested antibiotics. These results support the favorable safety profile of JM053 for potential food or probiotic applications.

#### 3.2.2. Hemolytic Activity

**Figure 5:**
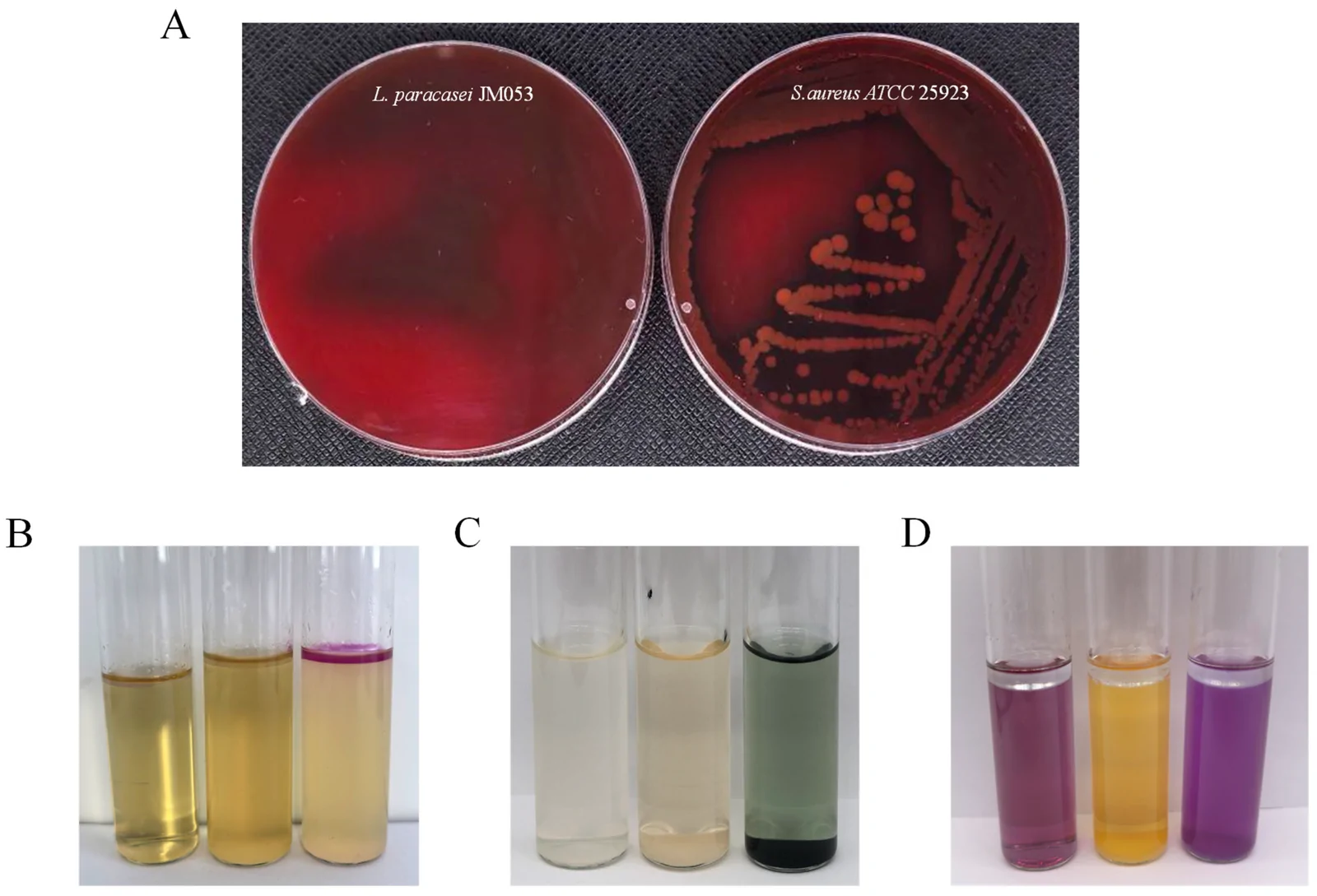
In vitro safety evaluation of *L. paracasei* JM053. (**A**) Hemolytic activity. (**B**) Indole test. (**C**) Nitrate reduction test. (**D**) Amino acid decarboxylase test. (**B**–**D**), from left to right: blank control, *L. paracasei* JM053, and *E. coli* ATCC 25922.

*L. paracasei* JM053 exhibited γ-hemolysis when cultured on Columbia blood agar, indicating the absence of hemolytic activity. In contrast, the control strain *S. aureus* ATCC 25923 displayed β-hemolysis (Figure 5A). According to the guidelines established by the FAO/WHO, probiotic strains must not exhibit hemolytic activity [52].

#### 3.2.3. Indole Detection

The indole test was employed to determine whether the strain produced tryptophanase, an enzyme that catabolizes tryptophan to yield a rosy-red indole product upon reaction with Kovac’s reagent. Tryptophan is an essential amino acid for humans, and disturbances in its metabolism can lead to functional disorders of the nervous, immune, and digestive systems [53]. As shown in Figure 5B, *L. paracasei* JM053 did not produce tryptophanase after incubation, whereas the positive control (*E. coli* ATCC 25922) formed a distinct rosy-red ring.

#### 3.2.4. Nitrate Reductase Activity

Nitrate reduction was assayed to evaluate nitrate reductase production, as the resulting nitrite is a potential carcinogen that can form nitrosamines with amines in vivo [54]. As shown in Figure 5C, a blue color indicated nitrate reductase activity: nitrate was reduced to nitrite, which reacted with potassium iodide to liberate iodine; the iodine-starch complex produced the characteristic blue. *L. paracasei* JM053 tested negative, showing no nitrate reductase activity.

#### 3.2.5. Amino Acid Decarboxylase Activity

The strain’s ability to produce biogenic amines via amino acid decarboxylation was assessed. Elevated biogenic amine levels pose health risks such as vomiting, hypertension, and headache [55]. In this assay, the alkaline medium containing bromocresol purple is purple; if no biogenic amines are formed, glucose fermentation alone produces acid, turning the indicator yellow. If biogenic amines are produced, they neutralize the acid, restoring the purple color. As shown in Figure 5D, *L. paracasei* JM053 remained yellow, confirming a negative result. Taken together, these findings demonstrate that *L. paracasei* JM053 possesses a favorable safety profile.

### 3.3. Genomic Analysis

#### 3.3.1. General Genome Characteristics

**Figure 6:**
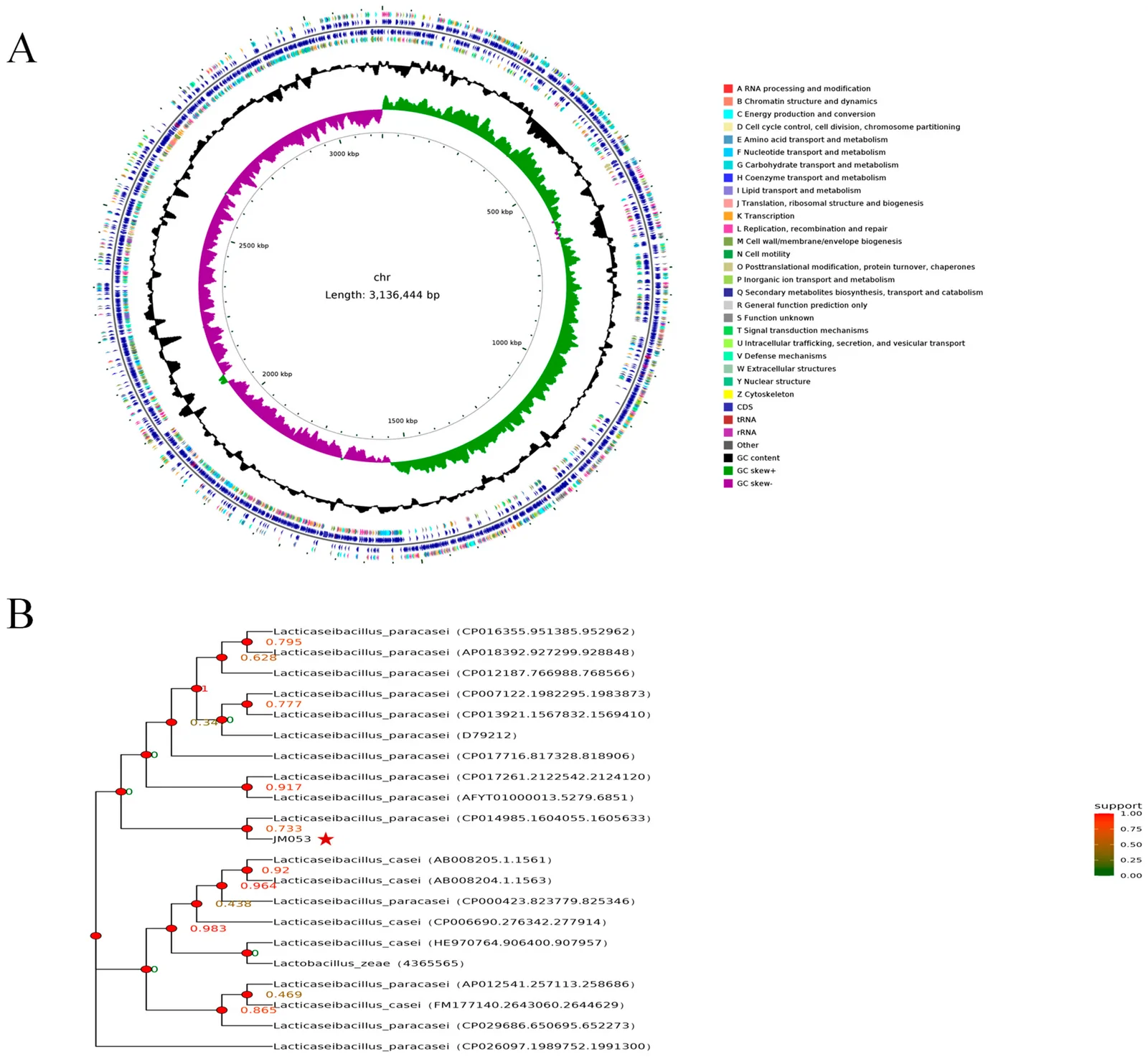
(**A**) Circular genome map of *L. paracasei* JM053, from the inside out, the first circle represents the scale; the second circle represents GC Skew; the third circle represents GC content; the fourth and seventh circles represent the COG to which each CDS belongs; the fifth and sixth circles represent the positions of CDS, tRNA, and rRNA on the genome; (**B**) Phylogenetic tree based on the 16S rDNA gene sequence of *L. paracasei* JM053. The red star in panel B indicates the position of strain JM053.

Whole-genome sequencing and bioinformatic analysis were performed to construct the complete genome map of *L. paracasei* JM053 (Figure 6A). The genome contained a circular chromosome measuring 3,136,444 bp, along with a plasmid of 3096 bp. The GC contents of the complete genome, chromosome, and plasmid were 46.32%, 46.33%, and 36.98%, respectively. The chromosome was predicted to harbor 3018 protein-coding genes, 59 tRNA genes, 15 rRNA genes, 30 genomic islands, 5 prophage regions, and 3 CRISPR elements, while the plasmid contained 3 protein-coding genes. The genome features of strain JM053 were generally consistent with those reported for the reference strain *L. paracasei* JCM8130 (GenBank: AP012541), including genome size, GC content, and the numbers of protein-coding genes, rRNA genes, and tRNA genes [56]. The 16S rRNA gene sequences of 20 closely related species were retrieved, and multiple sequence alignment was performed using MAFFT v7.5. A phylogenetic tree was then constructed with FastTree v2.1.11 (http://meta.microbesonline.org/fasttree/, accessed on 22 August 2026). As shown in Figure 6B, analysis of the 16S rRNA gene sequences revealed that strain JM053 clustered most closely with *Lacticaseibacillus paracasei*, further confirming its affiliation to this species.

#### 3.3.2. Coding Gene Prediction and Functional Annotation

**Figure 7:**
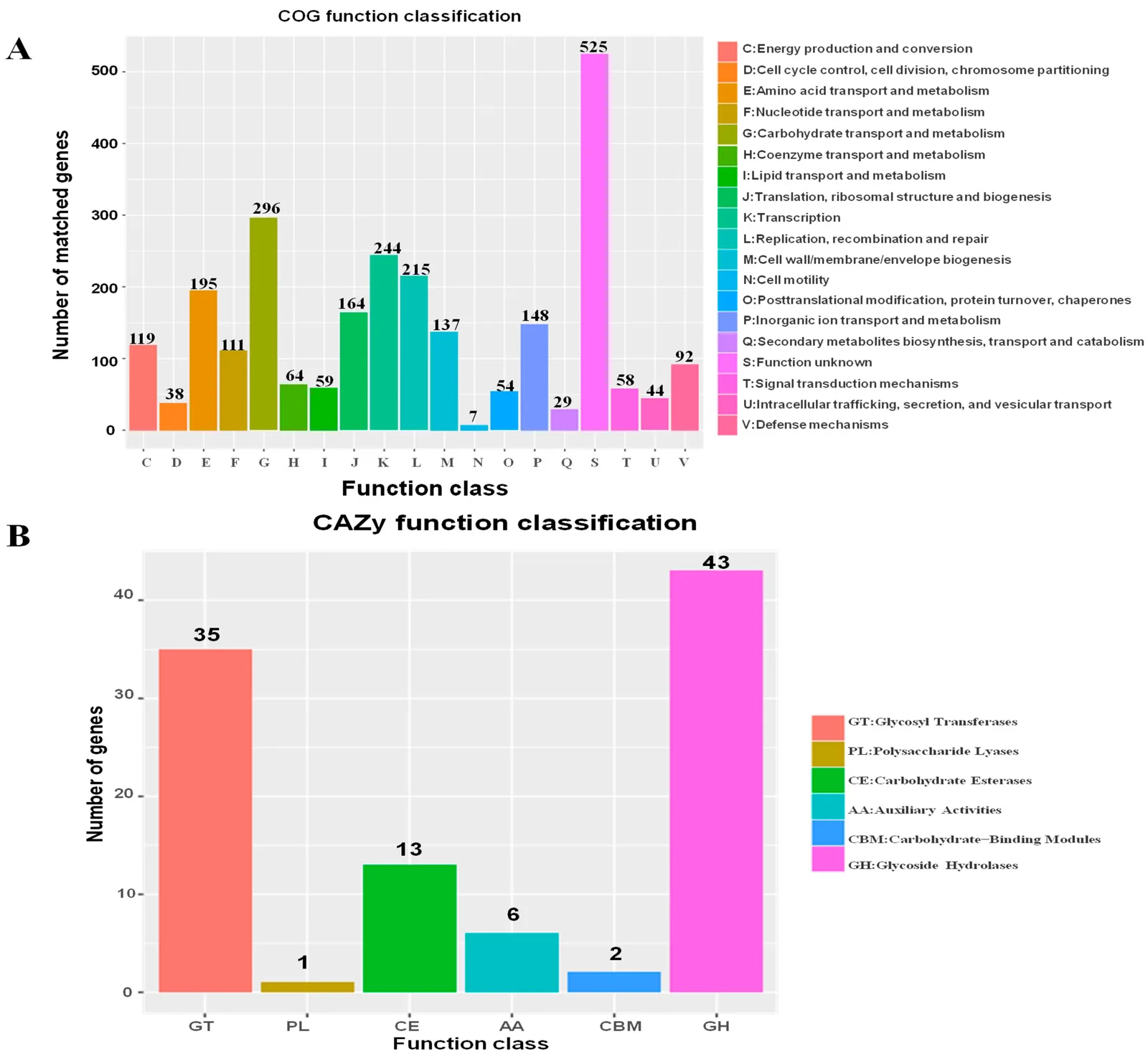
(**A**) COG database annotation of *L. paracasei* JM053; (**B**) CAZy database annotation of *L. paracasei* JM053.

Protein-coding genes were functionally annotated against the COG (Clusters of Orthologous Groups) database using eggNOG-mapper (Figure 7A). A total of 2599 genes were annotated, with the dominant functional categories being carbohydrate transport and metabolism (296 genes), transcription (244 genes), and amino acid transport and metabolism (195 genes). Additionally, genes linked to cell envelope biogenesis (137 genes) and energy metabolism (119 genes) were detected.

**Figure 8:**
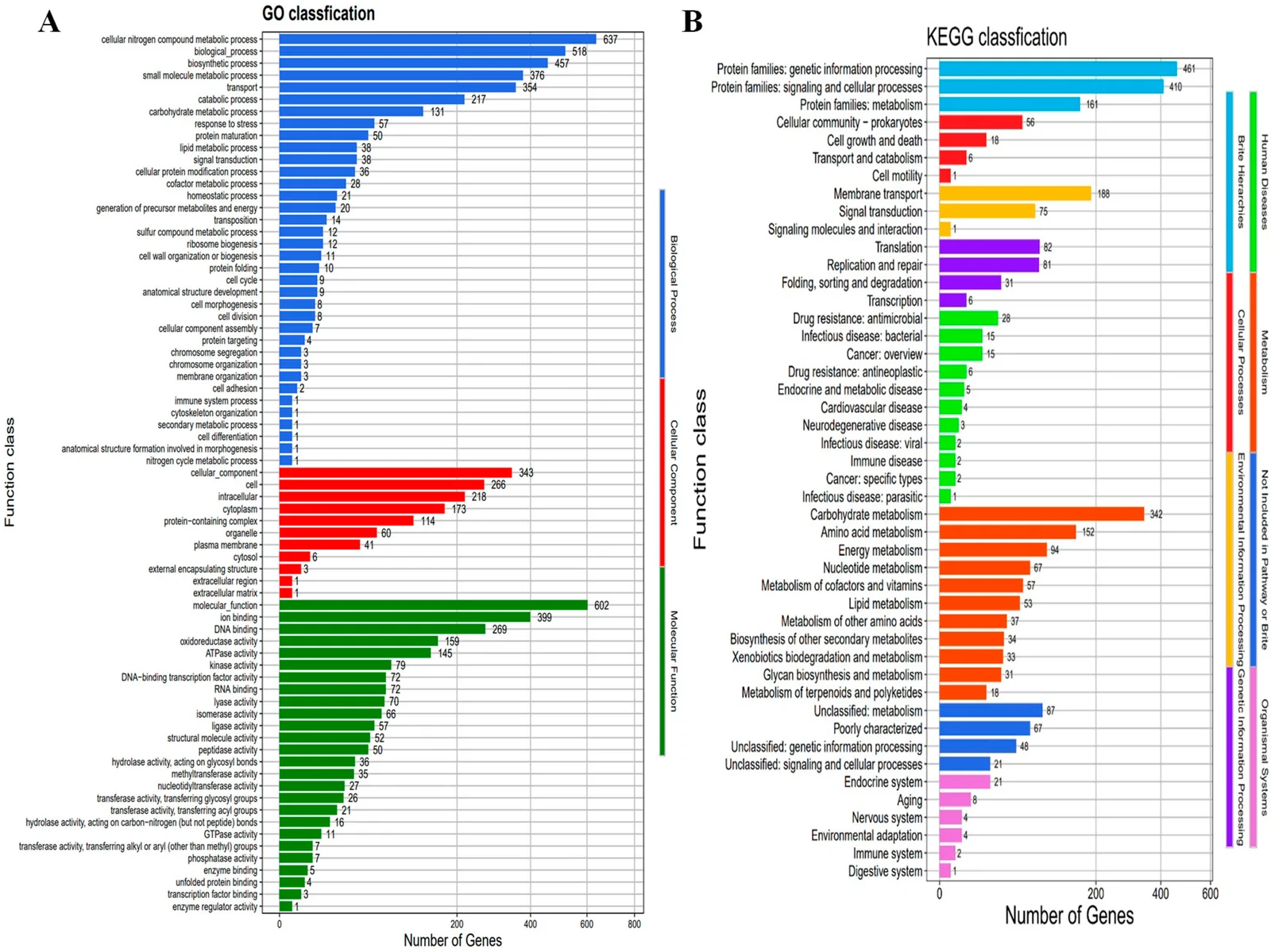
(**A**) GO database annotation of *L. paracasei* JM053; (**B**) KEGG database annotation of *L. paracasei* JM053.

Gene Ontology (GO) annotation of *L. paracasei* JM053 is shown in Figure 8A. Within the biological process category, genes involved in cellular nitrogen compound metabolic processes were the most abundant (637 genes) and were closely associated with protein metabolism. In the molecular function category, 50 genes encoding peptidase activity were identified. In the cellular component category, 41 genes were linked to the cell membrane, which is known to be involved in protein secretion and peptide transport.

In the KEGG annotation, 2841 genes of *L. paracasei* JM053 were assigned to functional categories (Figure 8B). The Brite Hierarchies category constituted the largest group (1032 genes), with enrichment in genetic information processing and metabolism. In the Metabolism category, 918 genes were annotated, including 342 involved in carbohydrate metabolism and 152 involved in amino acid metabolism, indicating the presence of pathways for carbon and nitrogen utilization.

Collectively, the genomic features observed here—particularly the abundance of genes associated with carbohydrate utilization, cell envelope components, and peptide metabolism—suggest a genetic predisposition of strain JM053 towards adhesion and proteolytic activities. Du et al. [19] showed that *L. paracasei* E1601-2 had significantly more genes involved in amino acid metabolism, membrane transport, and peptidase activity than the other strains tested, and experimentally validated its ability to hydrolyze β-lactoglobulin, further supporting the proteolytic potential of *L. paracasei*.

### 3.4. Correlation Analysis Between the Genomics of L. paracasei JM053 and Allergenic Epitopes in a Dual-Protein System

#### 3.4.1. Analysis of Proteolysis-Related Genes

Proteolysis in LAB is mediated by a coordinated proteolytic system consisting of cell-envelope proteinases (CEPs), peptide transporters, and intracellular peptidases. CEPs initiate the degradation of intact proteins into oligopeptides, which are subsequently transported into the cell and further hydrolyzed into smaller peptides and free amino acids [57]. Genome annotation revealed that *L. paracasei* JM053 possessed a complete proteolytic system, including the cell-envelope proteinase PrtP encoded by *prtP*, peptide transport systems (OppABCDF and DppABCDE), and diverse intracellular peptidases (Table 3 and Table 4). Following PrtP-mediated initial cleavage, oligopeptides can be transported into the cytoplasm through Opp and Dpp systems. Specifically, the Opp system consists of the substrate-binding protein OppA, transmembrane proteins OppB and OppC, and ATP-binding proteins OppD and OppF, and facilitates the uptake of peptides with different chain lengths [58].

After intracellular transport, peptides are further degraded by various peptidases, including endopeptidases, aminopeptidases, dipeptidases, and proline-specific peptidases. Notably, JM053 encoded proline-specific peptidases, including PepQ and PepX (Table 4). Due to the cyclic structure of proline, peptide bonds involving proline residues are relatively resistant to cleavage by many general peptidases. Proline-specific enzymes, such as PepQ and PepX, can efficiently hydrolyze proline-containing peptides and may contribute to more extensive degradation of complex protein substrates [59].

Considering that whey protein and soybean protein contain abundant proline residues and that some proline-containing sequences have been reported to be associated with allergenic epitopes, these proline-specific peptidases may enhance the degradation of proline-containing peptides during fermentation and potentially contribute to the reduction in protein allergenicity [60].

**Figure 9:**
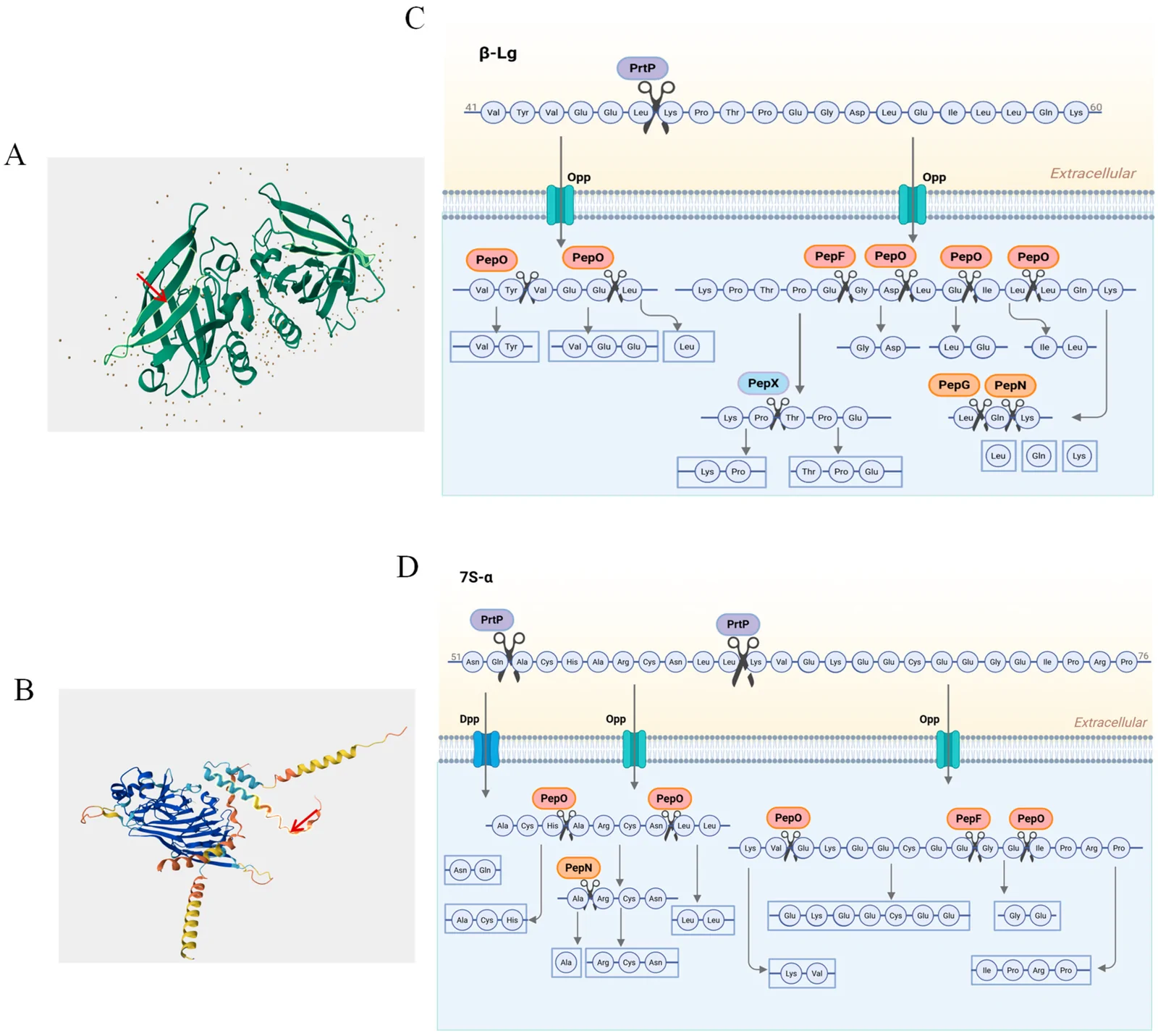
Correlation analysis between the genome of *L. paracasei* JM053 and allergenic epitopes. (**A**) 3D structure of β-Lg and allergenic peptide locations. (**B**) 3D structure of the 7S α-subunit and allergenic peptide locations. (**C**) Predicted proteolytic hydrolysis of allergenic peptide AA41-60. (**D**) Predicted proteolytic hydrolysis of allergenic peptide AA51-76. In panels (**C**,**D**), the arrows indicate the sequential hydrolysis direction of the peptide chains, and the different colors represent distinct proteases as well as different peptide transport systems involved in the hydrolysis and transport process. Panels (**C**,**D**) were created using BioRender.

To further elucidate the mechanism of allergenicity reduction, the cleavage patterns of two typical linear epitopes—β-Lg residues 41–60 and 7S α subunit residues 51–76—mediated by the proteolytic system of *L. paracasei* JM053 were predicted. The 3D structures of soybean 7S α subunit (UniProt P0DO16) and bovine β-Lg (UniProt P02754) were retrieved from UniProt (http://www.uniprot.org/, accessed on 22 August 2026) and the spatial distribution of their epitopes was annotated. Both epitopes were surface-exposed and displayed high structural flexibility and accessibility, rendering them readily recognized and bound by specific antibodies [61]. The β-Lg fragment (residues 41–60) spans both β-sheet and flexible loop regions [62], suggesting its allergenicity may involve both linear and conformational epitopes (Figure 9A). In contrast, the 7S α subunit fragment (residues 51–76) lies at the subunit terminus in an extended conformation and its allergenicity is primarily attributed to linear epitopes (Figure 9B). Therefore, it is hypothesized that proteolytic cleavage of linear epitopes during fermentation may represent a critical mechanism and an effective strategy for reducing protein allergenicity.

A cleavage-site-specific amino acid frequency matrix was constructed based on the MEROPS database, and a log-likelihood scoring method was applied to predict protease cleavage sites. As shown in Figure 9C, the β-Lg fragment AA41-60 is predicted to be fully hydrolyzed: PrtP is predicted to cleave extracellularly, the fragments are predicted to be imported via the Opp system, and endopeptidases (PepO, PepF) are predicted to further degrade them. PepX is predicted to specifically hydrolyze Pro-containing bonds (e.g., Pro-Thr), while PepN and PepG are predicted to release N-terminal amino acids. The predicted final products comprise two tripeptides, five dipeptides, and four free amino acids. Peptides shorter than five residues lose their allergenic potential [26]. As shown in Figure 9D, the 7S α subunit fragment AA51-76 is predicted to be completely degraded into one heptapeptide, one tetrapeptide, two tripeptides, four dipeptides, and one free amino acid. B-cell linear epitope prediction using the IEDB database indicated that all scores for the resulting heptapeptide “EKEECEE” were below the 0.5 threshold, suggesting its lack of allergenic potential. Overall, this in silico analysis provides a preliminary prediction of the mechanism by which *L. paracasei* JM053 disrupts linear epitopes through targeted enzymatic cleavage, offering a theoretical basis for allergenicity reduction by lactic acid bacterial fermentation.

#### 3.4.2. Analysis of Carbohydrate Metabolism-Related Genes

CAZy annotation identified 100 carbohydrate-active enzyme (CAZyme) genes in *L. paracasei* JM053, predominantly glycoside hydrolases (GHs, 43 genes) and glycosyltransferases (GTs, 35 genes) (Figure 7B). The prevalence of GH genes suggests a substantial genetic potential for carbohydrate metabolism, which may support energy supply for growth and potentially facilitate proteolysis [17], while the enrichment of GTs implies a notable capacity for glycosylation and polysaccharide synthesis [63].

**Figure 10:**
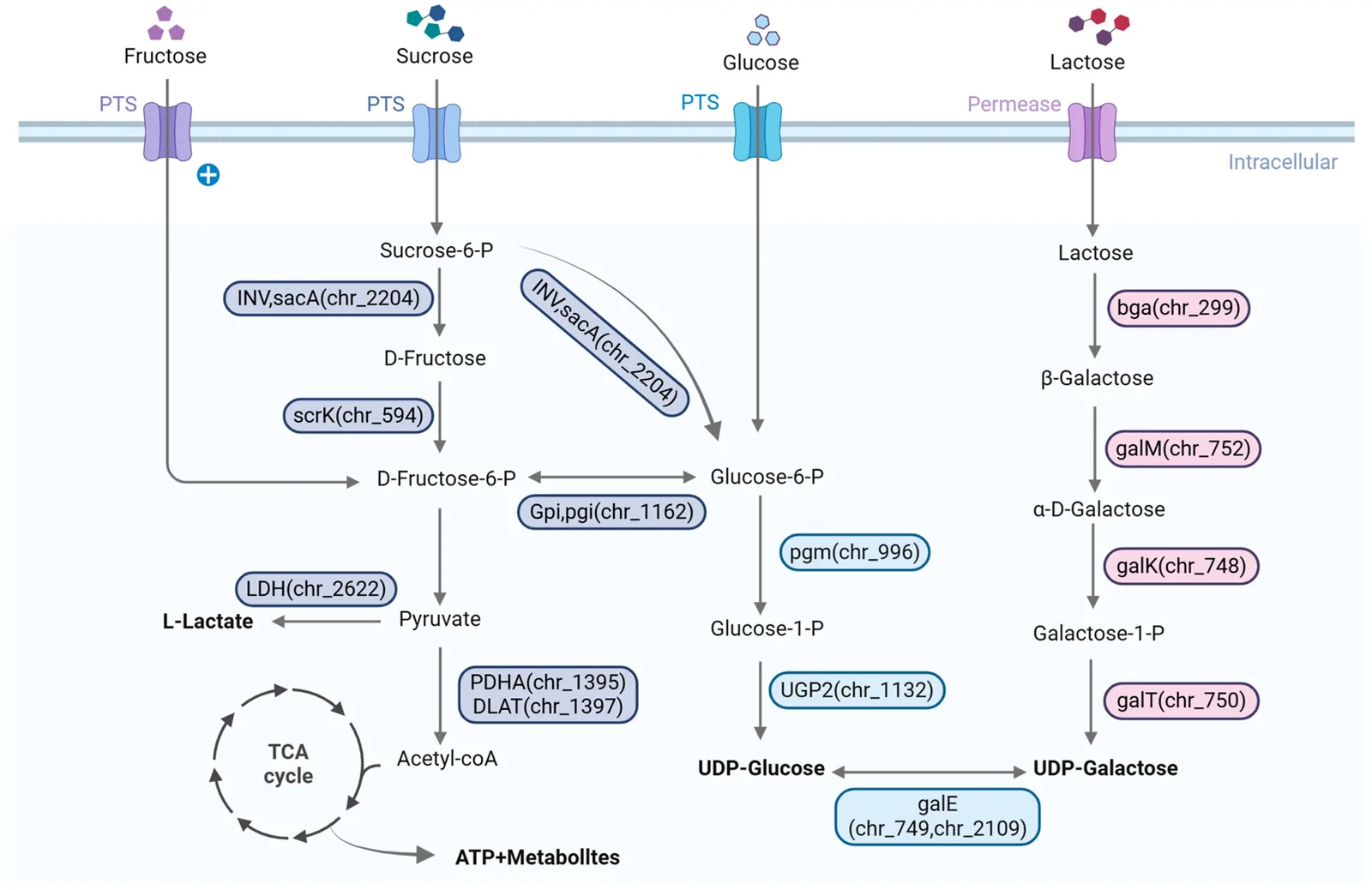
Predicted carbohydrate metabolism pathways in *L. paracasei* JM053. The arrows indicate the direction of metabolic flux in the pathway.

Based on the description of microbial sugar biosynthesis pathways by Liu et al. [17], we predicted the carbohydrate metabolic pathways of *L. paracasei* JM053, as illustrated in Figure 10. The genome prediction suggests that sucrose, glucose, and fructose are internalized via the phosphotransferase system (PTS; Table 4) and metabolized through glycolysis and the TCA cycle to generate ATP, supporting cellular growth (with potential energy implications for protease synthesis). Regarding lactose, the absence of a dedicated transporter suggests that it may enter cells via passive diffusion or broad-specificity permeases. After hydrolysis by β-galactosidase into glucose and galactose, the latter is predicted to be converted to UDP-galactose, which interconverts with UDP-glucose. These activated nucleotide sugars are likely to serve as glycosyl donors for glycosyltransferases. Overall, pathway analysis predicted that JM053 possesses complete genetic repertoires for sucrose, glucose, fructose, and lactose metabolism, indicating a strong genetic potential for carbon source utilization in the dual-protein system.

### 3.5. Biosynthetic Gene Clusters for Secondary Metabolites

**Figure 11:**
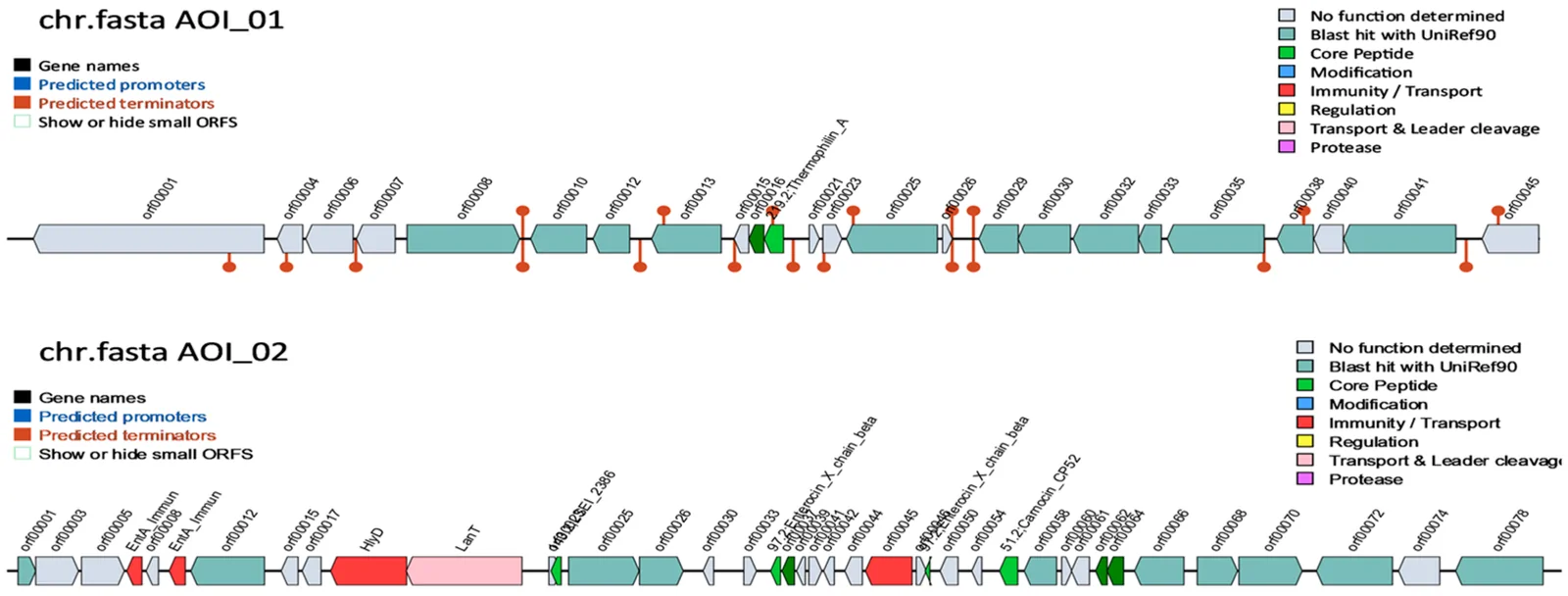
The biosynthetic gene cluster encoding antimicrobial compounds in the genome of *L. paracasei* JM053. Dark green indicates genes annotated as Bacteriocin_IIc by BAGEL4.

Functional genome analysis of *L. paracasei* JM053 via antiSMASH and BAGEL4 identified two predicted bacteriocin gene clusters (Figure 11). AOI_01 harbors a single gene encoding a class II bacteriocin core precursor peptide with 43.5% similarity to Thermophilin A from *Streptococcus thermophilus*; however, the absence of maturation and secretion elements suggests that this cluster is likely incomplete [64]. In contrast, AOI_02 contains a multi-bacteriocin gene cluster encoding several core peptides, including sequences homologous to the Enterocin X β-chain and Carnocin CP52. Notably, identical core peptides have been reported in the class II bacteriocin gene cluster of *L. paracasei* AD22 [65]. These genomic features indicate the genetic basis for potential bacteriocin production in JM053, although functional expression and inhibitory spectra require experimental confirmation [66].

### 3.6. Virulence Factor and Antibiotic Resistance Gene

VFDB analysis identified 18 putative virulence factor-associated genes on the chromosome of *L. paracasei* JM053, predominantly related to adhesion, stress survival, and immunomodulation (Table S4). Consistent with KEGG and COG annotations, these genes supported basic metabolism and normal growth without invasive traits [67].

Initial CARD analysis using default settings identified 32 antimicrobial resistance (AMR)-related sequence hits on the chromosome of *L. paracasei* JM053. To enhance reliability, these candidate hits were further evaluated according to the EFSA-recommended criteria of ≥80% sequence identity and ≥70% coverage of the subject sequence [68]. The sequence identity, coverage, and functional annotations of these hits were summarized in Table S5. None of these hits fulfilled the EFSA-recommended criteria for reporting AMR genes and were therefore not considered relevant AMR determinants. A similar finding was reported by Chen et al. in their genotypic safety assessment of *L. paracasei* subsp. *paracasei* NTU 101, where distant homologous resistance-related hits were also excluded after further screening [69]. Collectively, these genotypic findings are consistent with the phenotypic results, supporting that JM053 does not pose a safety concern regarding antibiotic resistance.

### 3.7. Analysis of Probiotic-Related Genes

Various stress conditions may affect the survival of lactic acid bacteria during processing and storage, as well as their subsequent colonization and functionality within the gastrointestinal tract. Consistent with this, the prediction of a substantial array of stress-response genes in JM053 under diverse conditions (Table S6) suggests that this strain may have a notable genetic capacity to cope with various environmental stresses, although functional validation would be required to confirm its tolerance phenotypes.

## 4. Conclusions

In this study, *L*. *paracasei* JM053 was identified as a promising fermentative strain with potential to reduce the in vitro allergenicity of the WPI-SPI dual-protein system. Among the 13 LAB strains evaluated, JM053 exhibited superior proteolytic performance and a high IgE-binding inhibition rate. Whole-genome analysis revealed that JM053 harbors a comprehensive proteolytic system, including PrtP, peptide transporters (OppABCDF and DppABCDE), and multiple intracellular peptidases, providing genomic evidence for its protein-degrading capability. Furthermore, in vitro safety evaluation and genomic analysis supported its favorable safety characteristics and probiotic potential. Overall, this study provides a promising candidate strain for the development of hypoallergenic dual-protein products and offers preliminary insights into the potential role of LAB-mediated proteolysis in allergenicity reduction.

It should be noted that the primary objective of this study was to screen LAB strains with potential proteolytic and allergenicity-reducing capabilities in the WPI-SPI dual-protein system rather than to elucidate fermentation kinetics. Therefore, the current study was designed based on a standardized 48 h fermentation endpoint, and the dynamic relationship between proteolysis and allergenicity reduction requires further investigation. In addition, acidified non-fermented controls and heat-inactivated cell controls were not included, which limits the discrimination of the contributions of acidification and cell-associated factors. Future studies incorporating time-course sampling and additional controls are needed to further clarify the mechanisms underlying LAB-mediated allergenicity reduction.

## Figures and Tables

**Table 1:** pH and viable cell counts of WPI-SPI fermented by different strains.

Sample	pH Value	Viable Lactic Acid Bacteria (×10^6^ CFU/mL)
unfermented WPI-SPI	7.00	
fermented WPI-SPI		
*L. paracasei* JY091	3.96 ± 0.42 ^e^	6.9 ± 0.52 ^e^
*L. paracasei* JM053	3.77 ± 0.64 ^fg^	7.4 ± 0.44 ^b^
*L. paracasei* JY062	3.86 ± 0.84 ^ef^	7.1 ± 0.35 ^cd^
*L. paracasei* JY56	3.58 ± 0.83 ^h^	6.2 ± 0.53 ^g^
*L. rhamnosus* JL-1	3.65 ± 0.54 ^gh^	6.5 ± 0.18 ^f^
*L. rhamnosus* JM039	3.68 ± 0.55 ^gh^	4.5 ± 0.39 ^h^
*L. rhamnosus* JY027	3.60 ± 0.71 ^h^	4.0 ± 0.49 ^i^
*L. gasseri* JM1	4.39 ± 0.42 ^bc^	6.9 ± 0.81 ^e^
*L. reuteri* J-1	4.50 ± 0.89 ^ab^	7.5 ± 0.14 ^b^
*L. plantarum* JY033	4.24 ± 0.39 ^cd^	7.2 ± 0.94 ^c^
*L. plantarum* JM065	4.20 ± 0.78 ^d^	7.0 ± 0.67 ^de^
*L. plantarum* J26	4.39 ± 0.99 ^bc^	7.5 ± 0.56 ^b^
*L. plantarum* JM015	4.56 ± 0.02 ^a^	7.8 ± 0.63 ^a^

Note: different letters within the same column indicate significant differences (*p* < 0.05).

**Table 2:** Types and contents of free amino acids in dual-protein fermented by different strains.

Species	Control	JY091	JM053	JY062	JY56	JL-1	JM039	JY027	JM1	J-1	J26	JY033	JM015	JM065
Asp	0.16 ± 0.04 ^k^	3.45 ± 0.58 ^g^	15.43 ± 1.17 ^a^	3.14 ± 0.52 ^h^	5.05 ± 0.41 ^e^	9.80 ± 1.37 ^c^	11.24 ± 0.49 ^b^	8.12 ± 0.88 ^d^	4.12 ± 0.82 ^f^	0.60 ± 0.09 ^j^	8.21 ± 0.52 ^d^	0.14 ± 0.03 ^k^	1.08 ± 0.14 ^i^	—
Pro	—	10.23 ± 0.30 ^i^	31.18 ± 1.44 ^a^	21.27 ± 2.02 ^e^	21.65 ± 1.32 ^d^	26.68 ± 2.12 ^b^	16.49 ± 0.99 ^f^	15.51 ± 1.53 ^g^	21.90 ± 1.20 ^c^	2.60 ± 0.65 ^k^	12.21 ± 0.96 ^h^	1.74 ± 0.19 ^l^	4.07 ± 0.16 ^j^	—
Asn	—	22.87 ± 1.38 ^h^	69.38 ± 0.94 ^a^	20.25 ± 2.60 ^i^	26.06 ± 1.29 ^g^	65.75 ± 3.05 ^b^	55.35 ± 5.04 ^c^	44.78 ± 4.30 ^d^	40.81 ± 3.16 ^e^	7.64 ± 1.22 ^j^	39.85 ± 2.05 ^f^	—	5.24 ± 0.71 ^k^	—
Arg	—	—	—	—	—	1.02 ± 0.25 ^c^	1.05 ± 0.06 ^c^	—	0.59 ± 0.06 ^d^	—	1.11 ± 0.17 ^c^	7.82 ± 0.37 ^a^	4.38 ± 0.36 ^b^	—
His	—	2.07 ± 0.55 ^g^	4.73 ± 0.64 ^d^	2.68 ± 0.55 ^f^	2.11 ± 0.46 ^g^	7.39 ± 1.30 ^a^	7.03 ± 0.70 ^b^	7.27 ± 0.60 ^a^	2.87 ± 0.30 ^e^	—	6.42 ± 1.00 ^c^	—	—	—
Lys	—	13.48 ± 0.72 ^f^	12.93 ± 2.16 ^g^	11.92 ± 1.15 ^h^	10.63 ± 0.83 ^i^	18.46 ± 1.84 ^c^	17.25 ± 1.15 ^d^	20.14 ± 1.04 ^a^	16.75 ± 1.17 ^e^	7.26 ± 0.84 ^k^	19.47 ± 0.67 ^b^	3.52 ± 0.22 ^l^	8.06 ± 0.56 ^j^	0.31 ± 0.04 ^m^
g-ABA	—	0.38 ± 0.09 ^b^	—	—	—	—	—	—	—	0.34 ± 0.11 ^b^	0.94 ± 0.16 ^a^	0.15 ± 0.04 ^c^	0.42 ± 0.09 ^b^	0.16 ± 0.03 ^c^
Phe	0.25 ± 0.08 ^kl^	1.04 ± 0.08 ^j^	11.39 ± 0.96 ^b^	2.30 ± 0.43 ^i^	4.11 ± 0.16 ^d^	3.47 ± 1.06 ^e^	3.17 ± 1.05 ^gh^	3.21 ± 0.70 ^fg^	6.00 ± 0.83 ^c^	0.30 ± 0.08 ^k^	3.35 ± 0.19 ^ef^	14.49 ± 1.26 ^a^	3.04 ± 0.19 ^h^	0.15 ± 0.02 ^l^
Tyr	—	—	—	—	—	—	—	—	0.49 ± 0.07 ^a^	—	—	—	—	—
Leu	—	12.66 ± 1.17 ^h^	24.29 ± 1.29 ^e^	6.03 ± 0.69 ^i^	13.01 ± 1.13 ^g^	35.59 ± 4.49 ^b^	36.12 ± 2.23 ^a^	28.89 ± 1.63 ^c^	19.67 ± 0.86 ^f^	1.46 ± 0.41 ^k^	26.82 ± 0.99 ^d^	—	3.66 ± 0.34 ^j^	—
Ile	—	8.01 ± 0.93 ^g^	20.49 ± 2.60 ^c^	4.75 ± 0.30 ^h^	13.23 ± 0.65 ^f^	24.99 ± 1.93 ^a^	24.68 ± 3.86 ^b^	13.18 ± 1.99 ^f^	16.58 ± 0.92 ^e^	—	17.53 ± 1.56 ^d^	—	2.98 ± 0.28 ^i^	—
Met	3.81 ± 0.54 ^h^	14.47 ± 1.08 ^e^	2.36 ± 0.41 ^i^	2.08 ± 0.50 ^j^	13.47 ± 0.78 ^f^	17.83 ± 0.37 ^c^	27.54 ± 3.15 ^a^	12.64 ± 2.55 ^g^	14.76 ± 1.65 ^d^	0.71 ± 0.07 ^l^	20.77 ± 2.01 ^b^	0.56 ± 0.06 ^m^	1.03 ± 0.13 ^k^	0.59 ± 0.07 ^lm^
Cys	9.22 ± 0.80 ^a^	—	—	—	—	—	—	—	—	0.39 ± 0.06 ^b^	—	—	—	—
Val	1.98 ± 0.40 ^k^	10.01 ± 0.42 ^i^	77.85 ± 1.10 ^a^	21.13 ± 1.10 ^h^	43.40 ± 3.01 ^d^	74.70 ± 4.99 ^b^	66.39 ± 3.56 ^c^	41.21 ± 5.29 ^e^	22.92 ± 2.48 ^g^	0.49 ± 0.10 ^m^	39.63 ± 3.81 ^f^	1.37 ± 0.07 ^l^	5.05 ± 0.58 ^j^	0.18 ± 0.02 ^n^
Ala	0.67 ± 0.17 ^k^	7.45 ± 0.62 ^h^	43.19 ± 0.87 ^a^	24.09 ± 0.78 ^d^	19.67 ± 2.13 ^e^	19.54 ± 2.00 ^e^	14.13 ± 4.51 ^g^	30.72 ± 3.88 ^b^	28.23 ± 2.75 ^c^	3.66 ± 0.47 ^i^	15.09 ± 0.76 ^f^	0.52 ± 0.09 ^l^	1.99 ± 0.42 ^j^	0.51 ± 0.06 ^l^
Gly	—	5.58 ± 0.41 ^i^	39.38 ± 1.84 ^a^	15.25 ± 0.89 ^g^	24.09 ± 0.90 ^d^	32.72 ± 3.58 ^c^	34.53 ± 5.14 ^b^	22.24 ± 2.64 ^e^	6.02 ± 0.66 ^h^	0.59 ± 0.08 ^k^	21.58 ± 0.54 ^f^	0.17 ± 0.02 ^l^	2.88 ± 0.15 ^j^	—
Gln	1.12 ± 0.32 ^l^	27.40 ± 2.36 ^h^	80.58 ± 4.53 ^a^	24.00 ± 1.25 ^i^	29.30 ± 1.77 ^g^	65.75 ± 5.43 ^c^	66.75 ± 6.01 ^b^	50.13 ± 8.35 ^e^	51.69 ± 3.05 ^d^	10.30 ± 0.86 ^j^	45.87 ± 3.37 ^f^	0.54 ± 0.10 ^m^	5.50 ± 0.53 ^k^	—
Glu	—	—	—	—	—	7.03 ± 0.31 ^a^	—	—	—	—	—	—	—	—
Ser	—	0.92 ± 0.24 ^i^	8.15 ± 0.86 ^d^	1.53 ± 0.48 ^h^	13.35 ± 2.73 ^c^	38.80 ± 2.82 ^a^	5.95 ± 0.98 ^e^	2.83 ± 0.54 ^f^	13.62 ± 1.31 ^b^	0.41 ± 0.07 ^k^	2.40 ± 0.06 ^g^	0.41 ± 0.02 ^k^	0.67 ± 0.09 ^j^	—
Thr	—	2.39 ± 0.26 ^g^	52.09 ± 3.66 ^a^	6.70 ± 0.74 ^f^	15.03 ± 1.34 ^d^	21.58 ± 1.41 ^b^	17.11 ± 1.68 ^c^	9.99 ± 0.72 ^e^	15.01 ± 1.08 ^d^	0.31 ± 0.09 ^i^	6.61 ± 0.57 ^f^	—	1.29 ± 0.13 ^h^	—
ΣEAAs	6.05 ± 0.86 ^m^	64.12 ± 3.61 ^h^	206.13 ± 6.81 ^a^	57.61 ± 3.23 ^i^	114.99 ± 3.49 ^f^	204.01 ± 7.23 ^b^	199.29 ± 5.27 ^c^	136.51 ± 6.91 ^e^	114.55 ± 5.09 ^g^	10.52 ± 1.31 ^l^	140.60 ± 5.08 ^d^	19.93 ± 1.55 ^k^	25.09 ± 2.03 ^j^	1.23 ± 0.09 ^n^
ΣTFAAs	17.22 ± 1.71 ^m^	142.40 ± 3.18 ^i^	493.42 ± 12.45 ^a^	167.13 ± 4.96 ^h^	254.16 ± 4.08 ^g^	471.10 ± 14.28 ^b^	404.80 ± 18.22 ^c^	310.85 ± 15.15 ^d^	282.01 ± 10.49 ^f^	37.05 ± 2.05 ^k^	287.86 ± 7.81 ^e^	31.43 ± 1.79 ^l^	51.31 ± 3.58 ^j^	1.90 ± 0.18 ^n^

Note: “—” indicates that the amino acid content was below the detection limit; ΣEAAs, total essential amino acids; ΣTFAA, total free amino acids; different letters in the same row indicate significant differences at *p* < 0.05.

**Table 3:** Peptide transport systems in *L. paracasei* JM053.

Gene ID	Gene Name	Gene Description
gene1695	*oppA*	MULTISPECIES: oligopeptide ABC transporter substrate-binding protein
gene1965	*oppA*	ABC transporter
gene1697, gene1964	*oppB*	MULTISPECIES: ABC transporter permease
gene1696, gene1963	*oppC*	MULTISPECIES: ABC transporter permease
gene1699	*oppD*	Oligopeptide transport ATP-binding protein OppD
gene1698, gene1960, gene2164	*oppF*	MULTISPECIES: ATP-binding cassette domain-containing protein
gene183, gene314, gene2168, gene2910	*dppA*	MULTISPECIES: peptide ABC transporter substrate-binding protein
gene2167	*dppB*	MULTISPECIES: ABC transporter permease
gene2166	*dppC*	MULTISPECIES: ABC transporter permease
gene1961, gene2165	*dppD*	ABC transporter ATP-binding protein
gene1300	*dppE*	MULTISPECIES: peptide ABC transporter substrate-binding protein

**Table 4:** Genes encoding proteolytic enzymes in *L. paracasei* JM053.

Protease	Genes	Cleavage Site Preference
S8 family serine peptidase	*prtP*	The P1 position is a hydrophobic amino acid (L/I/V/F/Y) or glutamine (Q)
Endopeptidase	*pepO*	Hydrophobic amino acids
	*pepF*	
Aminopeptidase	*pepN*	N-terminal neutral/hydrophobic/basic amino acids
	*pepC*	
	*pepG*	
	*pepS*	
	*pepT*	
Dipeptidase	*pepV*	Histidine-containing dipeptide, Xaa-His
	*pepDA*	
Proline peptidase	*pepQ*	The peptide bond between the first amino acid (Xaa) at the N-terminus and the second amino acid (proline, Pro)
	*pepX*	N-terminal Xaa-Pro dipeptide

## Data Availability

The original contributions presented in this study are included in the article/Supplementary Materials. Further inquiries can be directed to the corresponding authors.

## Supplementary Materials

The following supporting information can be downloaded at: https://www.mdpi.com/article/10.3390/foods15172947/s1, Table S1: Serum information of allergic patients; Table S2: Strain codes, species names, and sources; Table S3: Minimum Inhibitory Concentrations of Antibiotics for *L. paracasei* JM053; Table S4: Potential virulence factors of *L. paracasei* JM053; Table S5: CARD-derived antimicrobial resistance-related sequence hits identified in the genome of *L. paracasei* JM053; Table S6: Probiotic functional genes of *L. paracasei* JM053.
