# Supplementary material for: Screening of Fermentative Strains for Reducing the Allergenicity of a Whey Protein–Soy Protein System and Genomic Characterization of the Selected Strain
Source: Foods. 2026 Aug 22;15(17):2947. doi: 10.3390/foods15172947 (PMC13564616; doi:10.3390/foods15172947)
Supplement: Supplementary file 1 [file foods-15-02947-s001.zip › foods-4452984-supplementary.pdf]

Supplementary Materials:

Table S1. Serum information of allergic patients

Number	Allergen	Gender	Age	IgE level（kU/L）	Allergic symptoms
1	Bovine whey (α-La, β-Lg)	Female	21	4.81, 12.8	Allergic rhinitis
2	Bovine whey (α-La, β-Lg)	male	30	19.2, 12.5	Allergic rhinitis, asthma and dermatitis
3	Bovine whey (α-La, β-Lg)	Female	72	18.2, 3.61	Allergic rhinitis
4	Soybean	male	33	38.1	Allergic rhinitis
5	Soybean	male	32	6.83	Allergic rhinitis
6	Soybean	Female	34	4.5	Allergic rhinitis and asthma

Table S2. Strain codes, species names, and sources

Strain code	Species name	Source	Selection Rationale
JY091	*L. paracasei*	Tibetan traditional fermented dairy product	Previous studies have confirmed that the combination of this strain with royal jelly can enhance immune function in mice [23].
JM053	*L. paracasei*	Tibetan traditional fermented dairy product	Previous studies have confirmed that fermentation of soymilk with this strain promotes the release of small-molecular-weight protein peptides and enhances the antioxidant activity of the fermented soymilk [24].
JY062	*L. paracasei*	Tibetan traditional fermented dairy product	Previous studies have confirmed that this strain exhibits strong antioxidant activity and modulates intestinal immune function [25].
JY56	*L. paracasei*	Tibetan traditional fermented dairy product	Previous studies have reported that these types of LAB strains possess proteolytic activity or potential for allergenicity reduction [26].
JL-1	*L. rhamnosus*	Healthy infant feces	Previous studies have confirmed that this strain alleviates LPS-induced intestinal inflammation [27].
JM039	*L. rhamnosus*	Healthy infant feces	Previous studies have reported that these types of LAB strains possess proteolytic activity or potential for allergenicity reduction [28].
JY027	*L. rhamnosus*	Healthy infant feces	Previous studies have confirmed that this strain alleviates hyperuricemia in mice via the gut-kidney axis [29].
JM1	*L. gasseri*	Healthy infant feces	Previous studies have confirmed that fermentation of milk with this strain can improve the gut microbiota and promote gastrointestinal motility [30].
J-1	*L. reuteri*	Tibetan traditional fermented dairy product	Previous studies have reported that these types of LAB strains possess proteolytic activity or potential for allergenicity reduction [28].
J26	*L. plantarum*	Homemade fermented yogurt from Inner Mongolian herders	Previous studies have confirmed that this strain alleviates alcohol-induced liver inflammation [31].
JY033	*L. plantarum*	Tibetan traditional fermented dairy product	Previous studies have reported that these types of LAB strains possess proteolytic activity or potential for allergenicity reduction [17].
JM015	*L. plantarum*	Tibetan traditional fermented dairy product	Previous studies have reported that these types of LAB strains possess proteolytic activity

			or potential for allergenicity reduction [17].
JM065	*L. plantarum*	Tibetan traditional fermented dairy product	Previous studies have reported that these types of LAB strains possess proteolytic activity or potential for allergenicity reduction [17].

TableS3 Minimum Inhibitory Concentrations of Antibiotics for *L. paracasei* JM053

Antibiotic categories	Concentration range (μg/mL)										cut-off values（ug/mL）
Ampicillin	16-	8-	4-	2-	1-	0.5+	0.25+	0.125+	0.064+	0.032+	4
Gentamicin	128-	64-	32-	16-	8-	4+	2+	1+	0.5+	0.25+	32
Kanamycin	256-	128-	64-	32-	16+	8+	4+	2+	1+	0.5+	64
Streptomycin	256-	128-	64-	32-	16+	8+	4+	2+	1+	0.5+	64
Erythromycin	8-	4-	2-	1-	0.5-	0.25+	0.125+	0.064+	0.032+	0.016+	1
Clindamycin	16-	8-	4-	2-	1-	0.5-	0.25+	0.125+	0.064+	0.032+	4
Tetracycline	16-	8-	4-	2-	1-	0.5-	0.25-	0.125+	0.064+	0.032+	4
Chloramphenicol	16-	8-	4-	2-	1+	0.5+	0.25+	0.125+	0.064+	0.032+	4

Note: *Lacticaseibacillus paracasei* ATCC 334 was used as the quality-control strain, with acceptable MIC ranges based on ISO 10932 as reported previously [42].

Table S4. Potential virulence factors of *L. paracasei* JM053.

VFDB ID	Virulence factor	gene	description
VFG031960	Adherence	*lap*	aldehyde dehydrogenase (NAD) family protein
VFG016490		*tuf*	elongation factor Tu
VFG002165		*efaA*	zinc ABC transporter substrate-binding protein
VFG012103		*groEL*	chaperonin GroEL
VFG000077	Stress survival	*clpP*	ATP-dependent Clp endopeptidase proteolytic subunit ClpP
VFG000080		*clpE*	ATP-dependent Clp protease ATP-binding subunit
VFG000079		*clpC*	ATP-dependent Clp protease ATP-binding subunit
VFG005579	Exoenzyme	*eno*	phosphopyruvate hydratase
VFG026980	Regulation	*sigA/rpoV*	RNA polymerase sigma factor
VFG006826		*lisR*	two-component response regulator
VFG005871	Immune modulation	*hasC*	UTP--glucose-1-phosphate uridylyltransferase
VFG045688		*cpsA/uppS*	Undecaprenyl diphosphate synthase
VFG048851		*gndA*	NADP-dependent phosphogluconate dehydrogenase
VFG006089		*rfbD*	dTDP-4-dehydrorhamnose reductase
VFG006022		*rfbB*	dTDP-glucose 4,6-dehydratase
VFG005997		*rfbA*	glucose-1-phosphate thymidylyltransferase RfbA
VFG016416		*BT9727_RS 25850*	sugar transferase
VFG005999		*STU_RS14620*	UDP-N-acetylglucosamine--LPS N-acetylglucosamine transferase

Table S5. CARD-derived antimicrobial resistance-related sequence hits identified in the genome of *L. paracasei* JM053

ORF	ARO accession	CARD hit	Identity (%)	Coverage (%)	Annotation / mechanism	Assessment
chr_702	ARO:3000024	*patA*	51.773	100.000	ATP-binding cassette (ABC) antibiotic efflux pump; antibiotic efflux	Identity <80%; does not meet the EFSA general reporting criteria.
chr_715	ARO:3000300	*lsaA*	53.614	100.000	lsa-type ABC-F protein; antibiotic target protection	Identity <80%; does not meet the EFSA general reporting criteria.
chr_1764	ARO:3000838	*arlR*	53.744	100.000	major facilitator superfamily (MFS) antibiotic efflux pump; antibiotic efflux	Identity <80%; does not meet the EFSA general reporting criteria.
chr_2279	ARO:3002813	*lmrB*	46.638	96.646	ATP-binding cassette (ABC) antibiotic efflux pump; antibiotic efflux	Identity <80%; does not meet the EFSA general reporting criteria.
chr_703	ARO:3002882	*lmrD*	56.367	80.422	ATP-binding cassette (ABC) antibiotic efflux pump; antibiotic efflux	Identity <80%; does not meet the EFSA general reporting criteria.
chr_228	ARO:3002925	*vanR_in_vanF_cl*	49.123	97.835	glycopeptide resistance gene cluster; vanR; antibiotic target alteration	Identity <80%; does not meet the EFSA general reporting criteria.
chr_2920	ARO:3002928	*vanR_in_vanM_cl*	47.556	96.121	glycopeptide resistance gene cluster; vanR; antibiotic target alteration	Identity <80%; does not meet the EFSA general reporting criteria.
chr_1570	ARO:3003041	*Spne_PBP1a_AMX*	46.919	86.370	Penicillin-binding protein mutations conferring resistance to beta-lactam antibiotics; antibiotic target alteration	Identity <80%; does not meet the EFSA general reporting criteria.
chr_1750	ARO:3003078	*Efac_liaR_DAP*	70.531	98.095	daptomycin resistant liaR; antibiotic efflux; antibiotic target alteration	Identity <80%; does not meet the EFSA general reporting criteria.
chr_1554	ARO:3003092	*Efac_cls_DAP*	45.122	100.000	daptomycin resistant cls; antibiotic target alteration	Identity <80%; does not meet the EFSA general reporting criteria.
chr_2599	ARO:3003291	*Saur_rpoC_DAP*	69.224	98.094	daptomycin-resistant beta prime subunit of RNA polymerase (rpoC); antibiotic target alteration	Identity <80%; does not meet the EFSA general reporting criteria.
chr_7	ARO:3003296	*Saur_gyrA_FLO*	58.079	99.775	fluoroquinolone resistant gyrA; antibiotic target alteration	Identity <80%; does not meet the EFSA general reporting criteria.
chr_6	ARO:3003301	*Saur_gyrB_AMU*	62.381	97.667	aminocoumarin resistant gyrB; antibiotic target alteration	Identity <80%; does not meet the EFSA general reporting criteria.
chr_1497	ARO:3003311	*Spne_parC_FLO*	53.448	97.458	fluoroquinolone resistant parC; antibiotic target alteration	Identity <80%; does not meet the EFSA general reporting criteria.
chr_1496	ARO:3003314	*Saur_parE_AMU*	64.894	99.095	aminocoumarin resistant parE; antibiotic target alteration	Identity <80%; does not meet the EFSA general reporting criteria.
chr_2596	ARO:3003395	*Mtub_rpsL_STR*	62.5	99.194	antibiotic-resistant rpsL; antibiotic target alteration	Identity <80%; does not meet the EFSA general reporting criteria.
chr_1416	ARO:3003438	*Efac_EFTu_GE2A*	75.448	92.435	elfamycin resistant EF-Tu; antibiotic target alteration	Identity <80%; does not meet the EFSA general reporting criteria.

chr_1371	ARO:3003729	*Saur_ileS_MUP*	58.043	99.455	antibiotic-resistant isoleucyl-tRNA synthetase (ileS); antibiotic target alteration	Identity <80%; does not meet the EFSA general reporting criteria.
chr_2594	ARO:3003735	*Saur_fusA_FA*	70.851	99.567	antibiotic resistant fusA; antibiotic target alteration	Identity <80%; does not meet the EFSA general reporting criteria.
chr_2575	ARO:3003737	*Saur_fusE_FA*	57.303	100.000	antibiotic resistant fusE; antibiotic target alteration	Identity <80%; does not meet the EFSA general reporting criteria.
chr_2366	ARO:3003770	*Lmon_mprF*	46.996	97.803	defensin resistant mprF; antibiotic target alteration	Identity <80%; does not meet the EFSA general reporting criteria.
chr_967	ARO:3003788	*Bsub_pgsA_DAP*	48.958	100.000	daptomycin resistant pgsA; antibiotic target alteration	Identity <80%; does not meet the EFSA general reporting criteria.
chr_1751	ARO:3003791	*Efae_liaS_DAP*	45.763	95.368	daptomycin resistant liaS; antibiotic efflux; antibiotic target alteration	Identity <80%; does not meet the EFSA general reporting criteria.
chr_1845	ARO:3003899	*Ecol_PtsI_FOF*	48.342	99.304	antibiotic-resistant ptsI phosphotransferase; antibiotic target alteration	Identity <80%; does not meet the EFSA general reporting criteria.
chr_2590	ARO:3003930	*rpsJ*	67.647	99.029	tetracycline-resistant ribosomal protection protein; antibiotic target protection	Identity <80%; does not meet the EFSA general reporting criteria.
chr_1016	ARO:3003954	*efmA*	58.722	95.093	major facilitator superfamily (MFS) antibiotic efflux pump; antibiotic efflux	Identity <80%; does not meet the EFSA general reporting criteria.
chr_2214	ARO:3004049	*Ecol_fabG_TRC*	48.77	98.770	antibiotic resistant fabG; antibiotic target alteration	Identity <80%; does not meet the EFSA general reporting criteria.
chr_1470	ARO:3004153	*Mtub_thyA_PAS*	50.641	99.240	aminosalicylate resistant thymidylate synthase; antibiotic target alteration	Identity <80%; does not meet the EFSA general reporting criteria.
chr_2589	ARO:3005081	*Tthe_uL3_PLM*	46.19	99.515	Ribosomal protein mutation conferring resistance to pleuromutilin antibiotics; antibiotic target alteration	Identity <80%; does not meet the EFSA general reporting criteria.
chr_2600	ARO:3007073	*Bsub_rpoB_RIF*	71.513	98.994	rifamycin-resistant beta-subunit of RNA polymerase (rpoB); antibiotic target alteration; antibiotic target replacement	Identity <80%; does not meet the EFSA general reporting criteria.
chr_2573	ARO:3007527	*Bsub_rpsE_SPT*	72.05	96.988	spectinomycin resistant rpsE; antibiotic target alteration	Identity <80%; does not meet the EFSA general reporting criteria.
chr_1709	ARO:3007678	*IreK*	51.207	79.248	Serine/threonine kinases; reduced permeability to antibiotic	Identity <80%; does not meet the EFSA general reporting criteria.

Note: ORF, open reading frame; ARO, Antibiotic Resistance Ontology; CARD, Comprehensive Antibiotic Resistance Database. Identity represents the percentage of identical residues between the query and CARD reference sequences. Coverage represents the percentage of the CARD subject sequence covered by the alignment. According to the EFSA criteria, AMR-related sequence hits with ≥80% sequence identity and ≥70% subject sequence coverage should generally be reported.

Table S6. Probiotic functional genes of *L. paracasei* JM053.

Gene locus	Gene name	Gene function
Acid stress	*atpA*、*atpB*、*atpC*、*atpD*、*atpE*、*atpF*、*atpG*、*atpH*	ATP synthase
chr	*hisD*	Histidinol dehydrogenase
chr	*arcB*	ornithine cyclodeaminase
chr	*clpB*、*clpC*、*clpE*、*clpX*、*clpP*	ATP-dependent Clp protease
chr	*clcA*	ClC family H(+)/Cl(-) exchange transporter
Bile salt stress	*mdtG*	transporter, major facilitator family protein
chr	*ppaC*	manganese-dependent inorganic pyrophosphatase
chr	*dps*	DNA starvation/stationary phase protection protein
chr	*dltA*、*dltB*、*dltC*、*dltD*	D-alanine--poly(phosphoribitol) ligase subunit
Oxidative stress	*sodA*	superoxide dismutase
chr	*gshAB*	glutamate--cysteine ligase
chr	*trxA*	thioredoxin family protein
chr	*trxB*	thioredoxin-disulfide reductase
chr	*ahpC*	alkyl hydroperoxide reductase subunit C
chr	*perR*	Fur family transcriptional regulator
Osmotic stress	*opuCA*、*opuCB*、*opuCC*、*opuCD*	ABC transporter permease
chr	*nhaK*	sodium:proton antiporter
chr	*kup*	KUP/HAK/KT family potassium transporter
chr	*treA*	alpha,alpha-phosphotrehalase
chr	*treR*	trehalose operon repressor
chr	*gbuA*、*gbuB*、*gbuC*	glycine betaine/proline transport system permease
Temperature stress	*danK*、*danJ*、*grpE*、*groEL*、*groES*	molecular chaperone
chr	*csp*、*cspA*、*cspLA*	cold-shock protein
others	*hrcA*、*ctsR*	regulator
chr	*uvrA*、*uvrB*、*uvrC*、*recA*、*mutL*、*mutS*	DNA damage repair

References:

17. Liu, R.; Song, Y.; Li, Z.; Zhang, J.; Mu, G.; Jiang, S. Exploring the proteolytic activity of *Lactiplantibacillus plantarum* AHQ-14 to reduce the allergenicity of milk protein and its probiotic potential based on peptidomics and genomics. *J. Dairy Sci.* **2025**, *108*, 4573–4588. https://doi.org/10.3168/jds.2024-26245.

23. Ma, M.; Miao, C.; Zheng, M.; Hu, Z.; Zhang, Y.; Zhang, W.; Zhao, Q.; Jiang, Y. Effects of the complex of *Lactobacillus paracasei* JY091 and royal jelly on immunomodulatory effect and intestinal flora. *Food Biosci.* **2025**, *73*, 107623. https://doi.org/10.1016/j.fbio.2025.107623.

24. Zheng, Y.; Gao, K.; Li, Z.; Zhang, Y.; Li, X.; Zhang, W.; Guo, L.; Zhang, Y.; Jiang, Y. Fermentation of soymilk by strains with high conversion ability to soy isoflavones: Strain screening, flavor characteristics, antioxidant properties, and in vitro digestion. *Food Biosci.* **2025**, *66*, 106293. https://doi.org/10.1016/j.fbio.2025.106293.

25. Guo, J.; Sun, Y.; Gao, K.; Zhao, H.; Su, Y.; Zhao, Y.; Zhang, Y.; Jiang, Y. *Lacticaseibacillus paracasei* JY062 postbiotic alleviated 3% DSS-induced colitis in mice via integrated antioxidant, barrier repair,

immunomodulatory and microbiota modulation. *Antioxidants* **2025**, *14*, 1256. https://doi.org/10.3390/antiox14101256.
26. Xu, Y.; Zhang, F.; Ma, R.; Zhang, Z.; Chi, L.; Li, Y.; Xu, C.; Mu, G.; Zhu, X. Effects of *Lacticaseibacillus paracasei* XJ-003 on the allergenicity and antigenicity of milk proteins during fermentation. *Food Biosci.* **2024**, *59*, 103967. https://doi.org/10.1016/j.fbio.2024.103967.
27. Li, X.; Hu, D.; Tian, Y.; Song, Y.; Hou, Y.; Sun, L.; Zhang, Y.; Man, C.; Zhang, W.; Jiang, Y. Protective effects of a novel *Lactobacillus rhamnosus* strain with probiotic characteristics against lipopolysaccharide-induced intestinal inflammation in vitro and in vivo. *Food Funct.* **2020**, *11*, 5799–5814. https://doi.org/10.1039/d0fo00308e.
28. Begunova, A.V.; Savinova, O.S.; Glazunova, O.A.; Moiseenko, K.V.; Rozhkova, I.V.; Fedorova, T.V. Development of antioxidant and antihypertensive properties during growth of *Lactobacillus helveticus*, *Lactobacillus rhamnosus* and *Lactobacillus reuteri* on cow's milk: Fermentation and peptidomics study. *Foods* **2021**, *10*, 17. https://doi.org/10.3390/foods10010017.
29.0 Miao, C.; Dong, K.; Shen, Y.; Sun, Y.; Li, W.; Man, C.; Zhang, Y.; Zhao, Q.; Jiang, Y. Mechanism of *Lacticaseibacillus rhamnosus* JY027 alleviating hyperuricemia in mice through gut-kidney axis. *Food Biosci.* **2024**, *61*, 104757. https://doi.org/10.1016/j.fbio.2024.104757.
30. Cheng, S.; Li, B.; Ding, Y.; Hou, B.; Hung, W.; He, J.; Jiang, Y.; Zhang, Y.; Man, C. The probiotic fermented milk of *Lacticaseibacillus paracasei* JY062 and *Lactobacillus gasseri* JM1 alleviates constipation via improving gastrointestinal motility and gut microbiota. *J. Dairy Sci.* **2024**, *107*, 1857–1876. https://doi.org/10.3168/jds.2023-24154.
31. Li, H.; Cheng, S.; Huo, J.; Dong, K.; Ding, Y.; Man, C.; Zhang, Y.; Jiang, Y. *Lactobacillus plantarum* J26 alleviating alcohol-induced liver inflammation by maintaining the intestinal barrier and regulating MAPK signaling pathways. *Nutrients* **2023**, *15*, 190. https://doi.org/10.3390/nu15010190.
